# Supplementary material for: Prevalence of HIV, risk behaviours and vulnerabilities of female sex partners of the HIV positive people who inject drugs (PWID) in Dhaka city, Bangladesh
Source: PLoS One. 2023 Jun 5;18(6):e0286673. doi: 10.1371/journal.pone.0286673 (PMC10241362; doi:10.1371/journal.pone.0286673)
Supplement: S1 File — (PDF) [file pone.0286673.s001.pdf]

icddr,b

Annex 1: Consent Form for HIV positive men who inject drugs (MWID) (English)

**Unique ID**	☐☐☐☐☐	**MMT**	☐☐☐☐	**PWID**	☐☐☐☐

Protocol No.: **PR-19019**

Purpose of the study

I have come to you from icddr,b (cholera hospital, Mohakhali, Dhaka). My name is ________________. I know that you are HIV positive and have been receiving HIV prevention services from the Drop-In Centres (DICs) operated by Save the Children/Care Bangladesh. You may be aware that female sex partners (spouse, female sex workers and other female sex partners) of HIV positive People Who Inject Drugs (PWID) in Dhaka city are vulnerable to HIV infection. Hence, it is important to know if they are infected with HIV to ensure free treatment from the Government of Bangladesh that will prevent further development of severe illness.

Procedures of the study:

In this study, HIV will be tested using OraQuick on oral fluid that may take ~half an hour. If anyone found positive during OraQuick, WHO three rapid tests at icddr,b will be used to test for HIV by drawing blood of 5ml (one tea spoon full). After HIV testing by OraQuick, another 30-40 minutes time will be taken to collect information on socio-demographics, injecting and sexual risk behaviours, vulnerabilities to HIV and receiving HIV prevention services. The test and interview will be conducted maintaining privacy at the residence or any suitable place or at the DIC where ever the respondent feels comfortable. Children up to 17 years of age will also be tested only if the mother is found HIV positive and if you and your spouse/female sex partner agrees. Children who are less than 2 years old, viral nucleic acid will be tested to determine HIV by taking blood of 5ml and for children 2-17 years of age OraQuick will be used to test for HIV.

Before HIV testing, a female counsellor will take written informed consent from her. If the result is negative, result will be given on the spot and post-test counselling will be conducted. After confirmatory

HIV testing (if needed), post-test counselling will be conducted with her if found positive, she will be referred to the implementing partners for care, support and treatment (CST) services with free of cost.

What is expected from you?

Given the current situation of HIV among PWID in Dhaka city, it is urgently necessary to conduct HIV testing among all female sex partners of HIV positive male PWID. Therefore, we are expecting that you would kindly grant permission by fulfilling a written consent so that we can recruit your female sex partner (spouse/FSW/other female sex partner) in this study.

Risk and benefits

If she is found HIV positive during OraQuick we will need to take blood of 5ml and all aseptic precautions will be taken. This is a harmless procedure and is associated only with the mild discomfort of drawing blood. If your wife/transactional sex partner/non-transactional sex partner tested positive then your HIV status should be disclosed to them for ensuring free treatment to both as per government rules. We expect that data from this study will be useful in enhancing uptake of HIV tests that will allow treatment to be made available to those who are HIV positive. She will receive indirect benefit from this study as the data generated from this study will be used to guide policy and prevention activities for HIV in the country for the female sex partners of HIV positive male PWID.

Privacy, anonymity and confidentiality

Participation and all information given by your female sex partner will be kept strictly confidential. Testing for HIV using OraQuick and risk behaviour interview will be done in a private place at home or at DIC or at a suitable place where ever you feel comfortable.

Future use of information

We will store aliquots of whole blood and serum sample for 5 years for possible use in the future for further tests of hepatitis C virus detection and HIV drug resistance if adequate funding is available. Samples will be stored at the Virology Laboratory of icddr,b and will remain under the custodianship of the same Laboratory. All these stored samples will only have age, sex, study name and random ID number; no other information will be recorded in the labels.

Right not to participate and withdraw

Participation in this study is solely voluntary. She may choose not to answer any or all questions that will be asked and to not provide a sample of saliva or blood. She can leave the study at any time even in the middle of an interview. She has the right to refuse participation in this study, which will not affect existing HIV prevention services and treatment facilities that are being provided for you.

Principle of compensation

Treatment for HIV positive individuals is absolutely free. We will provide the respondent, accompanied by you, conveyance allowance for coming to the DIC for this study purpose, if needed. We will also provide you conveyance allowance for taking us to the respondent. We will also provide a small refreshment for the respondent at the end of the interview.

Persons of Contact

Please feel free to ask any questions that you may have. If you think of any questions later, you may contact the principal investigator of this study, Md. Masud Reza of icddr,b, Mohakhali, Dhaka, phone: 9827001-10, extension: 4205.

If you kindly agree to let your female sex partner/children to participate in our study, please indicate that by putting your signature or your left thumb impression at the specified space below.

Thank you very much for your cooperation.

___________________________________ ____________________

Signature or left thumb impression of HIV positive PWID Date

___________________________________ ___________________

Signature of the PI or his representative Date

If disagree, write details:

icddr,b

Annex 2: Consent Form for female sex partner of HIV positive MWID (English)

(For those who are 18 years of above)

Unique ID | | | | | |

Protocol No.: **PR-19019**

Population Group	Code (Circle, when applicable)
Spouse	1
FSW	2
Other female sex partner (except spouse and FSW) of **married** HIV positive PWID	3
Other female sex partner (except spouse and FSW) of **unmarried** HIV positive PWID	4

Purpose of the study

I have come to you from icddr,b (cholera hospital, Mohakhali, Dhaka). My name is ________________. You may be aware that female sex partners (spouse, female sex workers and other female sex partners) and children of HIV positive People Who Inject Drugs (PWID) in Dhaka city are vulnerable to HIV infection. Hence, it is important to know if they are infected with HIV to ensure free treatment from the Government of Bangladesh that will prevent further development of severe illness.

Procedures of the study:

In this study, HIV will be tested using OraQuick on oral fluid that may take ~half an hour. If you are found positive during OraQuick, WHO three rapid tests at icddr,b will be used to test for HIV by drawing blood of 5ml (one tea spoon full). After HIV testing, another 30-40 minutes of time will be taken to collect information on socio-demographics, injecting, sexual risk behaviours, vulnerabilities and receiving HIV prevention services from NGOs. The test and interview will be conducted maintaining privacy at the residence or any suitable place or at the DIC where ever you feel comfortable. Children up to 17 years of age will also be included if you agree. Children who are less than 2 years old, viral nucleic acid will be tested to determine HIV by taking blood of 5ml (one tea spoon full) and for children 2-17 years of age OraQuick will be used to test for HIV.

Before HIV testing, a female counsellor will take written informed consent from you. If the test result is negative, result will be provided on the spot and post-test counselling will be conducted. After confirmatory HIV testing (if needed), post-test counselling will be conducted with you and then you will be referred to the implementing partners for care, support and treatment (CST) services with free of cost.

What is expected from you?

Given the current situation of HIV among PWID in Dhaka city, it is urgently necessary to conduct HIV testing among all female sex partners of HIV positive PWID and their children up to age 17 years. Therefore, we are expecting that you would kindly agree to take part in this study.

Risk and benefits

If you are found HIV positive during OraQuick we will need to take blood of 5ml and all aseptic precautions will be taken. This is a harmless procedure and is associated only with the mild discomfort of drawing blood. If your testing result is positive then your HIV status should be disclosed to your husband/guardian for ensuring free treatment as per government rules. We expect that data from this study will be useful in enhancing uptake of HIV tests that will allow treatment to be made available to those who are HIV positive. You will receive indirect benefit from this study as the data generated from this study will be used to guide policy and prevention activities for HIV in the country for the female sex partners of male HIV positive PWID.

Privacy, anonymity and confidentiality

Participation and all information given by you will be kept strictly confidential. Testing for HIV using OraQuick and risk behaviour interview will be done in a private place at your home or at DIC or at a suitable place where ever you feel comfortable.

Future use of information

We will store aliquots of your whole blood and serum sample for 5 years for possible use in the future for further tests of hepatitis C and HIV drug resistance if adequate funding is available. Samples will be stored at the Virology Laboratory of icddr,b and will remain under the custodianship of the same Laboratory. All these stored samples will only have age, sex, study name and random ID number; no other information will be recorded in the labels.

Right not to participate and withdraw

Participation in this study is solely voluntary. You may choose not to answer any or all questions that will be asked and to not provide a sample of saliva or blood. You can leave the study at any time even in the middle of an interview. You have the right to refuse participation in this study, which will not affect existing treatment facilities at the DICs and outreach that your husband/boyfriend/sex partner has been receiving.

Principle of compensation

Treatment for HIV positive individuals is absolutely free. We will provide conveyance allowance accompanied by your spouse/anyone you prefer for going to the DIC for this study purpose, if needed. We will also provide a small refreshment at the end of the interview.

Persons of Contact

Please feel free to ask any questions that you may have. If you think of any questions later, you may contact the principal investigator of this study, Md. Masud Reza of icddr,b, Mohakhali, Dhaka, phone: 9827001-10, extension: 4205.

If you kindly agree to participate or let your child up to 17 years of age to participate in our study, please indicate that by putting your signature or your left thumb impression at the specified space below.

Thank you very much for your cooperation.

___________________________________ ____________________

Signature or left thumb impression of the respondent Date

___________________________________ ___________________

Signature of the PI or his representative Date

If disagree, write details:

Annex 3: Assent Form for female sex partner of HIV positive MWID (English)

(For those who are 15 to less than 18 years)

Unique ID | | | | | |

Protocol No.: **PR-19019**

Population Group	Code (Circle, when applicable)
Spouse	1
FSW	2
Other female sex partner (except spouse and FSW) of **married** HIV positive PWID	3
Other female sex partner (except spouse and FSW) of **unmarried** HIV positive PWID	4

Purpose of the study

I have come to you from icddr,b (cholera hospital, Mohakhali, Dhaka). My name is ________________. You may be aware that female sex partners (spouse, female sex workers and other female sex partners) and children of HIV positive People Who Inject Drugs (PWID) in Dhaka city are vulnerable to HIV infection. Hence, it is important to know if they are infected with HIV to ensure free treatment from the Government of Bangladesh that will prevent further development of severe illness.

Procedures of the study:

In this study, HIV will be tested using OraQuick on oral fluid that may take ~half an hour. If you are found positive during OraQuick, WHO three rapid tests at icddr,b will be used to test for HIV by drawing blood of 5ml (one tea spoon full). After HIV testing, another 30-40 minutes of time will be taken to collect information on socio-demographics, injecting, sexual risk behaviours, vulnerabilities and receiving HIV prevention services from NGOs. The test and interview will be conducted maintaining privacy at the residence or any suitable place or at the DIC where ever you feel comfortable. Children up to 17 years of age will also be included if you agree. Children who are less than 2 years old, viral nucleic acid will be tested to determine HIV by taking blood of 5ml (one tea spoon full) and for children 2-17 years of age OraQuick will be used to test for HIV.

Before HIV testing, a female counsellor will take written informed consent from you. If the test result is negative, result will be provided on the spot and post-test counselling will be conducted. After confirmatory HIV testing (if needed), post-test counselling will be conducted with you and then you will be referred to the implementing partners for care, support and treatment (CST) services with free of cost.

What is expected from you?

Given the current situation of HIV among MWID in Dhaka city, it is urgently necessary to conduct HIV testing among all female sex partners of HIV positive MWID and their children up to age 17 years. Therefore, we are expecting that you would kindly agree to take part in this study.

Risk and benefits

If you are found HIV positive during OraQuick we will need to take blood of 5ml and all aseptic precautions will be taken. This is a harmless procedure and is associated only with the mild discomfort of drawing blood. If your testing result is positive then your HIV status should be disclosed to your husband/guardian for ensuring free treatment as per government rules. We expect that data from this study will be useful in enhancing uptake of HIV tests that will allow treatment to be made available to those who are HIV positive. You will receive indirect benefit from this study as the data generated from this study will be used to guide policy and prevention activities for HIV in the country for the female sex partners of male HIV positive PWID.

Privacy, anonymity and confidentiality

Participation and all information given by you will be kept strictly confidential. Testing for HIV using OraQuick and risk behaviour interview will be done in a private place at your home or at DIC or at a suitable place where ever you feel comfortable.

Future use of information

We will store aliquots of your whole blood and serum sample for 5 years for possible use in the future for further tests of hepatitis C and HIV drug resistance if adequate funding is available. Samples will be stored at the Virology Laboratory of icddr,b and will remain under the custodianship of the same Laboratory. All these stored samples will only have age, sex, study name and random ID number; no other information will be recorded in the labels.

Right not to participate and withdraw

Participation in this study is solely voluntary. You may choose not to answer any or all questions that will be asked and to not provide a sample of saliva or blood. You can leave the study at any time even in the middle of an interview. You have the right to refuse participation in this study, which will not affect existing treatment facilities at the DICs and outreach that your husband/boyfriend/sex partner has been receiving.

Principle of compensation

Treatment for HIV positive individuals is absolutely free. We will provide conveyance allowance accompanied by your spouse/anyone you prefer for going to the DIC for this study purpose, if needed. We will also provide a small refreshment at the end of the interview.

Persons of Contact

Please feel free to ask any questions that you may have. If you think of any questions later, you may contact the principal investigator of this study, Md. Masud Reza of icddr,b, Mohakhali, Dhaka, phone: 9827001-10, extension: 4205.

If you kindly agree to participate or let your child up to 17 years of age to participate in our study, please indicate that by putting your signature or your left thumb impression at the specified space below.

Thank you very much for your cooperation.

____________________________________ _____________________

Signature or left thumb impression of the participant Date

Consent of the Guardian

Do you agree to allow this woman to participate in this study, if 'Yes' then please indicate that by putting your signature or your left thumb impression at the specified space below

____ _____________________

Signature or left thumb impression of Guardian Date

____________________________________ ____________________

Signature of the PI or his representative Date

If disagree, write details:

Annex 4: Pre-test Counselling form for female sex partner of HIV positive male MWID (English)

Unique ID | | | | | |

**Assessment of personal coping strategies:**	
	(Please circle code)
Client indicates history of depression	Prior history 1 Current history 2 None 3
Client indicates history of anxiety	Prior history 1 Current history 2 None 3
Client indicates history of other psychiatric disorder	Prior history 1 Current history 2 None 3
Client indicates suicide intent if test result is HIV positive	Yes 1 No 2
Client has prior history of suicide attempt	Yes 1 No 2
Client has prior history of self-harm attempt	Yes 1 No 2
Client indicates intent to harm another if test result is HIV positive	Yes 1 No 2
Client indicates potential risk of violence if discloses to sexual partner	Yes 1 No 2<br>Specify:
Client has adequate personal support network	Yes 1 No 2 Specify:

Certification from counsellor during pre-test counselling *(Please tick appropriate boxes)*

I certify that the following activities were performed during this counselling session:

- ☐ Build rapport and introduce your role to the client, explain about service and record keeping
- ☐ The confidentiality and privacy that you can offer the client
- ☐ Assess client's readiness to learn sero status
- ☐ Exploration of what the client might do if the test is positive, and the possible ways of coping with a HIV-positive result. This may include a suicide risk assessment if indicated.
- ☐ Exploration of potential support from family and friends
- ☐ Basic information about the test and result provision procedure

<u>Other notes (if needed):</u>

________________________ ____________________ __________________

Name of Counsellor Signature of Counsellor Date:

Annex 5: Quantitative questionnaire to assess HIV risk behaviours and vulnerabilities among female sex partner of HIV positive MWID, 2019 (English)

Unique ID | | | | | |

Risk behaviours questionnaire of female sex partner of HIV positive male PWID,2019

Population Group	Code (Tick, when applicable)
Spouse	1
FSW	2
Other female sex partner (except spouse and FSW) of **married** HIV positive PWID	3
Other female sex partner (except FSW) of **unmarried** HIV positive PWID	4

Name of Thana: ------------------------------------ Thana code: [ ]

Interviewer's code: [ ] Interviewer's name: ………………………………………………….

Date of Interview: -- (DD/MM/YYYY)

Interview starting time: ------------------------------ (HH:MM) (24HR)

Interview ending time: ------------------------------- (HH:MM) (24HR)

Has the interview been completed?

1. Yes
2. No

If no, why (only one answer allowed): …………………………………………………...………………….

Checked by the supervisor: Signature: - - - - - - - - - - - - - - - - - - - Date: -------------- (DD/MM/YYYY)

Supervisor's code: [ ]

Section 1: Background Characteristics
(Ask this section to all)

Q. No.	Questions	Coding categories	Skip to	Comments
101	How old are you?<br>(In completed years)	----------Years		
102	How many years of education have you completed up to now?	Never been to school 99<br># Years Completed _____<br>Less than one year 00<br>Only can signature 96<br>Don't know/Can't remember 97<br>No response 98		
103	In what type of area do you live currently most of the time?	Residential area 1<br>Slum 2<br>On the street 3<br>Others ………… 4		
104	Currently with whom do you live most of the time?	Alone 1<br>With relatives 2<br>With friends 3<br>No fixed address (on the street) 4<br>With PWID friends 5<br>With PWID husband 6<br>Other 7<br>Specify______________________		
105	What was the approximate total expenditure in your household in the last month?	Tk. _______<br>Don't know/Can't remember 97<br>No response 98		
106	What do you do for earning money?<br><br>(Do not read out)<br>(Multiple answers possible)<br>**(Provide ranking)**	Ranking<br>House wife 1 -----------<br>Running a grocery shop 2 ----------<br>Work at garments 3 ----------<br>Work at house-holds 4 ----------<br>Work in shop 5-----------<br>Sex work 6 ----------<br>Others .... 10 ----------<br>Don't know/Can't remember 97<br>No response 98		

Section 2: Marriage and sexual partnership

(Ask this section to all)

Q. No.	Questions	Coding categories	Skip to	Comments
201	What is your current marital status?	Married 1<br>Unmarried 2<br>Divorced 3<br>Widow 4<br>Separated 5<br>Widower 6<br>No Response 98	→ **207**	
202	How old were you when you got married first time/last time?	Age in years ____<br>Don't know/Can't remember 97<br>No Response 98		
203	Currently living with spouse?	Yes 1<br>No 2		
204	How many living children do you have currently?	Zero.....0<br>Number______<br>No response 98	→**206**	
205	How old is your youngest child?<br>(In completed years)	1st Child: Year.......Month......<br>2nd Child: Year.......Month......<br>3rd Child: Year.......Month......<br>14th Child: Year.......Month......		
206	Are you currently pregnant?	Yes 1<br>No 2<br>How many month..........		
207	At what age did you first have sexual intercourse? **(vaginal)**	Age in years ____<br>Don't know/Can't remember 97<br>No Response 98		
208	Had you used condom during last vaginal sex?	Yes 1<br>No 2<br>Never used condom 3<br>Don't know/Can't remember 97<br>No Response 98		
209	Had you taken any blood for any reason in the last 5 years?	Yes 1<br>No 2<br>Don't know/Can't remember 97<br>No Response 98		
210	If yes, what was the reason?			
211	Do you have history of tattooing?	Yes 1<br>No 2		

Section 3: Drug and injection related risk behaviours

(Ask this section to all)

No.	Questions and Filters	Coding categories	Skip to	Comments
301	Some people take drugs for fun or to get high. Have you taken any such drugs in the last 12 months?	Yes 1<br>No 2<br>Don't know/Can't remember 97<br>No Response 98	306	
302	If yes, mention the name of drugs taken in the last 12 months?<br><br>**(Do not read out)**<br>**(Multiple answers possible)**<br>**(If yes, circle 1)**<br>**(If no, circle 2)**	Sleeping pill 1 2<br>Cannabis 1 2<br>Alcohol 1 2<br>Phensidyl 1 2<br>Heroin 1 2<br>Buprenorphine/Pethidine (Injection) 1 2<br>Yaba 1 2<br>Others ------------------- 1 2		
303a	When did you last take drugs?	Within a month 00<br>Months ago____<br>Don't know/Can't remember 97<br>No Response 98		
303b	How long have you been taking any kind of drugs?	Years ---------<br>Months ----------<br>Within a month 0_<br>Don't know/Can't remember 97<br>No Response 98		
304	Why do you take drugs?	1.---<br>2.---<br>3.---		
305	Who inspired you to take drugs?	1.---<br>2.---<br>3.---		
306	Some people have tried injecting drugs for fun or to get high. Have you injected drugs in the last 12 months?	Yes 1<br>No 2<br>Don't know/Can't remember 97<br>No Response 98	 **→319**<br>**→ 319**<br>**→ 319**	
307	How long have you been injecting drugs?	Years ---------____Months ----------_<br>Don't know/Can't remember 97<br>No Response 98		
308	When did you last inject drugs?	Within a month 00<br>Months ago____<br>Don't know/Can't remember 97<br>No Response 98		

No.	Questions and Filters	Coding categories	Skip to	Comments
309	How many times had you taken injections in last 7 days?	Zero 0<br>Number____<br>Don't know/Can't remember 97<br>No Response 98		
310	How many times share injections in last 7 days?	Zero 0<br>Number of taken........<br>Number of given........<br>Don't know/Can't remember 97<br>No Response 98		
311	Why do you inject drugs?	1.-------------------------------------<br>2. ------------------------------------<br>3. ------------------------------------		
312	Who inspired you to inject drugs?	1.-------------------------------------<br>2. ------------------------------------<br>3. ------------------------------------		
313	Last time (within last 12 months) you injected, did you use a needle or syringe after your husband/boyfriend/sex partner had used it before?	Yes 1<br>No 2<br>Don't know/Can't remember 97<br>No Response 98		
314	Last time (within last 12 months) you injected, did you pass your used needle or syringe to your husband/boyfriend/sex partner?	Yes 1<br>No 2<br>Don't know/Can't remember 97<br>No Response 98		
315	Last time (within last 12 months) you injected, did you use a needle or syringe after anyone **except** your husband/boyfriend/sex partner had used it before?	Yes 1<br>No 2<br>Don't know/Can't remember 97<br>No Response 98		
316	Last time (within last 12 months) you injected, did you pass your used needle or syringe to anyone **except** your husband/boyfriend/sex partner?	Yes 1<br>No 2<br>Don't know/Can't remember 97<br>No Response 98		
317	Do you know of any place where you can obtain new needles and syringes?	Yes 1<br>No 2<br>Don't know/Can't remember 97<br>No Response 98	} 316	
318	Where can you obtain new syringes and needles?<br><br>**DO NOT READ OUT**<br>**Multiple answer possible**<br>**Circle 1 if mentioned**<br>**Circle 2 if not mentioned**	Pharmacy 1 2<br>Friend 1 2<br>Fellow Drug User 1 2<br>NGO worker 1 2<br>Drug Seller 1 2<br>Other (specify).............1 2<br>Don't know/Can't remember 97		

No.	Questions and Filters	Coding categories	Skip to	Comments
		No Response 98		
319	Are you currently involved with OST?	Yes 1<br>No 2<br>Don't know/Can't remember 97<br>No Response 98	401	
320	If yes, how long you have been involved?	Months………<br>Within a month 0<br>Don't know/Can't remember 97<br>No Response 98		

Section 4: Sexual behaviour with spouse

(Ask this section to only those females who are currently married)
(Let's talk about sex with your spouse, so far you can remember)

Q. No.	Questions	Coding categories	Skip to	Comments
401	When did you have your last sex with your spouse?	Within 1 month 0<br>------ months ago<br>Unmarried 96<br>Don't know/Can't remember 97<br>No Response 98	→ **404**	
402a	How many times vaginal sex with your spouse in last 12 months?	Within 1 month 0<br>------ months ago<br>Don't know/Can't remember 97<br>No Response 98		
402b	In what frequency you have used condoms during vaginal sex with your spouse in the last 12 months? **(Read out options 1-3)**	Always 1<br>Sometimes 2<br>Never 3<br>No response 98		
403	Have you used condoms during vaginal sex with your spouse in the last 12 months?	Yes 1<br>No 2<br>Don't know/Can't remember 97<br>No Response 98		
404	Did you have oral sex with your spouse in last 12 months?	Till ejaculation 1<br>Before ejaculation 2<br>Never 3<br>Don't know/Can't remember …97<br>No response 98	**406**	
405	If yes, how often did you use condoms last 12 months?	Always 1<br>Sometimes 2<br>Never 3<br>No response 98		

Q. No.	Questions	Coding categories	Skip to	Comments
406	Had you anal sex with your spouse in last 12 months?	Yes 1<br>No 2<br>Don't know/Can't remember 97<br>**No Response 98**	**408**	
407	If yes, how often did you use condoms during last 12 months? **(Read out options 1-3)**	Always 1<br>Sometimes 2<br>Never 3<br>No response 98		
408	Do you know if your husband injects drugs for fun or to get high?	Yes 1<br>No 2<br>Don't know/Can't remember 97<br>**No Response 98**		

Section 5: Sexual behaviour of female sex workers (FSW)

(Ask this section to those who are FSW)
(Let's talk about sex with a male sex partner, so far you can remember)

Q. No.	Questions	Coding categories	Skip to	Comments
501	How many frequencies did you have vaginal sex with male clients in the last 12 months?	Within 1 month 0<br>------ months ago<br>Within a week 96<br>Don't know/Can't remember 97<br>No Response 98<br>Never 99		
502	How many frequencies did you have vaginal sex with male clients in the last 12 months by exchange of money?	Number ....<br>Don't know/Can't remember 97<br>No Response 98		
503	How many times did you have vaginal sex with male clients in last 12 months?	Within 1 month 0<br>Don't know/Can't remember 97<br>No Response 98	→ **506**	
504	In what frequency you have used condoms during vaginal sex with male sex partners in the last 12 months? **(Read out options 1-3)**	Always 1<br>Sometimes 2<br>Never 3<br>Never sex in last 12 months 96<br>No response 98		
505	Did you use a condom the last time you had vaginal sex with your male sex partner in the last 12 months?	Yes 1<br>No.....2<br>Did not had sex in last 12 months 96<br>Don't know/Can't remember 97<br>No response 98		
506	Did you have oral sex till ejaculation with your male sex partners in last 12 months?	Till ejaculation 1<br>Before ejaculation 2<br>Never 3	→ **508**	

Q. No.	Questions	Coding categories	Skip to	Comments
		Never sex in last 12 months 96<br>Don't know/Can't remember 97<br>No response 98		
507	If yes, how often did you use condoms in last 12 months?<br>**(Read out options 1-3)**	Always 1<br>Sometimes 2<br>Never 3<br>No response 98		
508	Did you have anal sex with your male sex partners in last 12 months?	Yes 1<br>No 2<br>Never sex in last 12 months 96<br>Don't know/Can't remember 97<br>No response 98	**→ 510**	
509	If yes, how often did you use condoms in last 12 months?<br>**(Read out options 1-3)**	Always 1<br>Sometimes 2<br>Never 3<br>No response 98		
510	Do you know if any of your clients (new or regular) inject drugs for fun or to get high?	Yes 1<br>No 2<br>Don't know/Can't remember 97<br>No response 98		

Section 6: Sexual behaviour of non-transactional female sex partners
(Ask this section to those who are neither spouse nor FSW)
(Let's talk about sex with a male sex partner, so far you can remember)

Q. No.	Questions	Coding categories	Skip to	Comments
601	When did you have your last sex with a male sex partner?	Within 1 month 0<br>------ months ago<br>No 2<br>Don't know/Can't remember 97<br>No Response 98<br>Never 99		
602	How many times did you have vaginal sex with non commercial sex partners?	Zero 0<br>Number ------<br>Don't know/Can't remember 97<br>No Response 98		
603	In what frequency you have used condoms during vaginal sex with male sex partners in the last 12 months??<br>**(Read out options 1-3)**	Always 1<br>Sometimes 2<br>Never 3<br>No response 98		
604	Did you use a condom the last time you had vaginal sex with your male sex partner in the last 12 months?	Yes 1<br>No.....2<br>Don't know/Can't remember ...97<br>No response 98		

Q. No.	Questions	Coding categories	Skip to	Comments
605	Did you have oral sex till ejaculation with your male sex partners during last 12 months?	Till ejaculation 1<br>Before ejaculation 2<br>Never 3<br>Don't know/Can't remember 97<br>No response 98	→ **607**	
606	If yes, how often did you use condoms during last 12 months? **(Read out options 1-3)**	Always 1<br>Sometimes 2<br>Never 3<br>No response 98		
607	Did you have anal sex with your male sex partners during last 12 months?	Yes 1<br>No.....2<br>Don't know/Can't remember ...97<br>No response 98	→ **609**	
608	If yes, how often did you use condoms during last 12 months? **(Read out options 1-3)**	Always 1<br>Sometimes 2<br>Never 3<br>No response 98		
609	Do you know if your non-transactional sex partner injects drugs for fun or to get high?	Yes 1<br>No 2<br>Don't know/Can't remember 97<br>No response 98		

Section 7: Knowledge and treatment of STDs
(Ask this section to all)
(Now, I will ask something about STDs)

Q. No.	Questions	Coding categories	Skip to	Comments
701	Could you describe any symptoms in women of diseases that can be transmitted by having sex (vaginal/anal/oral)?<br><br>**DO NOT READ OUT**<br><br>**Circle 1 if mentioned**<br>**Circle 2 if not mentioned**<br>(Multiple responses possible)	Vaginal discharge 1 2<br>Smelly discharge 1 2<br>Genital ulcers/sores 1 2<br>Lower abdominal pain 1 2<br>Other ……………… 1 2<br>Don't know/Can't remember 97<br>No response 98		
702	In the last year, have you had a painful or smelly discharge from your vagina?	Yes 1<br>No 2<br>Don't know/Can't remember 97<br>No Response 98		
703	In the last year, have you had pain in your lower stomach area that was not associated with your period or a stomach upset?	Yes 1<br>No 2<br>Don't know/Can't remember 97<br>No Response 98		

Q. No.	Questions	Coding categories	Skip to	Comments
704	In the last year, have you had warts, sores or ulcers in your genital area?	Yes 1<br>No 2<br>Don't know/Can't remember 97<br>No Response 98		
705	CHECK ANSWERS TO Q 702, 703, 704, **If yes in any of these circle-1 otherwise circle-2**	Any yes........1<br>All no..........2	 → **801**	
706	Are you currently taking any treatment?	Yes 1<br>No 2<br>Don't know/Can't remember 97<br>No Response 98	 → **801**	
707	If yes, where?<br><br>DO NOT READ OUT<br>(Only one response)	Treatment from Gov. hospital...... 1<br>Treatment from pharmacy...... 2<br>Treatment from private doctor...... 3<br>Treatment from private clinic......4<br>Treatment from NGO clinic......5<br><br>Name of NGO Clinic____________<br><br>Treatment from traditional healer......6<br>Advice/treatment from friend.......7<br>Self treatment......... 8<br>Other__________________ 9<br>Don't know/Can't remember 97<br>No Response 98		

Section 8: Knowledge about HIV
(Ask this question to all)
(Now, I will ask about HIV)

Q. No.	Questions	Coding categories	Skip to	Comments
801	Have you ever heard of HIV?	Yes 1<br>No 2<br>Tested from this DIC 3<br>No response 98	 → **901**<br>→ **901**<br>→ **901**	
802	Can people reduce their risk of HIV by using a condom correctly every time they have vaginal sex?	Yes 1<br>No 2<br>Don't know/Can't remember ......97<br>No response 98		
803	Can a person get HIV from mosquito bites?	Yes 1<br>No 2<br>Don't know/Can't remember ......97<br>No response 98		
804	Can a person get HIV by sharing a meal with someone who is infected?	Yes 1<br>No 2<br>Don't know/Can't remember ......97<br>No response 98		

Q. No.	Questions	Coding categories	Skip to	Comments
805	Can a person get HIV by taking injections with a needle/syringe that was already used by someone else?	Yes 1<br>No 2<br>Don't know/Can't remember ……97<br>No response 98		
806	Can people reduce their risk of HIV by avoiding sex with multiple sex partners?	Yes 1<br>No 2<br>Don't know/Can't remember ……97<br>No response 98		
807	Do you think you can tell by looking at someone whether they are infected with HIV?	Yes 1<br>No 2<br>Don't know/Can't remember ……97<br>No response 98		

Section 9: Knowledge and uptake of confidential HIV testing

(Ask this section to all)
(Now, I will ask about confidential HIV testing)

Q. No.	Questions	Coding categories	Skip to	Comments
901	Do you know anywhere you could go if you want to get a confidential test to find out if you are infected with HIV/AIDS?	Yes 1<br>No 2<br>Tested from this DIC 3<br>Don't know/Can't remember ……97<br>No response 98	**→ 1001**<br><br>**→ 1001**<br>**→ 1001**	
902	**I don't want to know the result**, but have you ever had an HIV test?	Yes 1<br>No 2<br>Don't know/Can't remember ……97<br>No response 98	**→ 904**<br>**903**	
903	Why did you not seek HIV testing?<br><br>**(Multiple responses possible)**<br>**(DO NOT READ OUT)**<br>**Circle 1 if mentioned**<br>**Circle 2 if not mentioned**<br><br>**(After asking this question, go to 905)**	No one told me to test<br>Fear or concern of stigma by family members<br>Fear or concern of stigma by NGO staff<br>Fear or concern of stigma by neighbours<br>Fear or concern or experienced violence/police harassment or arrest<br>Others…………..	1 2<br>1 2<br>1 2<br>1 2<br>1 2<br>1 2	1001
904	If yes, where the HIV test was conducted last time?			
905	In the last time, did you yourself request the test or someone advised you to test for HIV or were you required to have the test?	Self 1<br>Someone advised 2<br>Required 3<br>No response 98		
906	Please do not tell me the result did you get the result of your test?	Yes 1<br>No 2<br>Don't know/Can't remember ……97	1001	

Q. No.	Questions	Coding categories	Skip to	Comments
		No response 98		
907	When did you have the most recent HIV test?	Within last six months 0<br>More than six months to one year ……1<br>More than a year ago ………2<br>Don't know/Can't remember 97<br>No response 98		

Section 10: HIV risk perceptions
(Ask this section to all)
(Now, I will ask about HIV risk perceptions)

Q. No.	Questions	Coding categories	Skip to	Comments
1001	Do you think that you are at risk for HIV?	High risk……..1<br>Some risk……..2<br>Little risk……..3<br>No risk……..4<br>HIV Positive.......96<br>Don't know 97<br>No response……98	→ 1002<br>→ 1002<br>→ 1003<br>→ 1003<br>→ 1101<br>→ 1101<br>→ 1101	
1002	Why do you think you are at **high or some** risk for HIV?<br><br>**(DO NOT READ OUT)**<br>**(Multiple answers possible)**<br>**(Circle 1 if mentioned)**<br>**(Circle 2 if not mentioned)**	Risky behaviour ……. 1 2<br>Frequent anal sex …….. 1 2<br>Frequent vaginal sex …….. 1 2<br>Irregular condom use ….. 1 2<br>Shared needles/syringes ……….. 1 2<br>Other-------------------------------- 1 2<br>Don't know/Can't remember 97<br>No response……98	If answer is any one not need to ask 1003 question	
1003	Why do you think you are at **little or no risk** of HIV?<br><br>**(DO NOT READ OUT)**<br>**(Multiple answers possible)**<br>**(Circle 1 if mentioned)**<br>**(Circle 2 if not mentioned)**	Always use condoms ……..1 2<br>Partners are clean ……….1 2<br>Partners are healthy.......1 2<br>Never share needles/syringes 1 2<br>Sometimes share needles/syringes 1 2<br>Irregular use of condom 1 2<br>Always have sex with single trusted partner 1 2<br>Always be neat and clean 1 2<br>Do less sex 1 2<br>Wash after sex work 1 2<br>Other____________________1 2<br>Don't know/Can't remember 97<br>No response 98		

Section 11: Knowledge about Hepatitis C
(Ask this section to all)
(Now, I will ask about knowledge of Hepatitis C)

Q. No.	Questions	Coding categories	Skip to	Comments
1101	Have you ever heard of Hepatitis C virus?	Yes 1<br>No 2<br>No response 98	→ **1201**	
1102	If yes, please mention what is the source of information?	1.---------------------------------- Rank-1<br>2. --------------------------------- Rank-2<br>3. --------------------------------- Rank-3		

Section 12: Exposure to HIV prevention services
(Now, I will ask about HIV prevention services)

Q. No.	Questions	Coding categories	Skip to	Comments
1201	Have you **ever** received HIV prevention services from NGO?	Yes 1<br>No 2<br>Don't know/Can't remember 97<br>No response 98	**1301**	
1202	When did you last receive HIV prevention services from any NGO?	Within in a month 0<br>……………. months ago<br>Don't know/Can't remember 97<br>No response 98		
1203	In your whole life which type of services have you received from NGO/Self-help group/CBO/Gov hospital or clinic?<br><br>**(Do not read out)**<br>**(Multiple answers possible)**<br>**(Circle 1 if mentioned)**<br>**(Circle 2 if not mentioned)**	Needle/syringe 1 2<br>General health education 1 2<br>Health education on HIV/STI/HCV 1 2<br>Condoms 1 2<br>Treatment for STD 1 2<br>Treatment for general health 1 2<br>Attending ANC 1 2<br>Received ART 1 2<br>Sleeping 1 2<br>Watching TV/Playing Ludu 1 2<br>Other ……………………….1 2<br>Don't know/Can't remember 97<br>No Response 98		

Section 13: History of TB Treatment
(Now, I will ask about TB Treatment)

Sl#	Questions	Types of code		Direction	Comments
1301	Have you ever diagnosed with TB?	Yes (Tested)<br>No (Tested)<br>Never tested<br>Don't know<br>No responses	1<br>2<br>3<br>97<br>98	→ 1401	
1302	If yes, how many times?	Times	----		
1303	If yes, type of TB	Pulmonary TB (Lung TB)<br>Extra pulmonary TB (TB anywhere except lung) (Specify site ....)	1<br>2		
1304	Have you received treatment for TB?	Yes<br>No<br>Don't know<br>No responses	1<br>2<br>97<br>98	1401	
1305	If yes, most recent treatment regimen?	Category 1 (6 months course of oral anti TB drug)<br>Category 2 (8 months course of oral anti TB drug including 2 months of injectable drug)<br>Others (Specify) --------------	1<br>2<br>3		
1306	If yes, what was the outcome?	Treatment ongoing<br>Cured (Treatment completed and tested negative)<br>Treatment completed (Treatment completed but test not yet done)<br>Treatment failure (Treatment completed but tested positive)<br>Lost to follow-up (Did not start treatment or stop medicine for >=2 months)<br>Others (Specify) -----------	1<br>2<br>3<br>4<br>5<br>6		
1307	From where did you get your treatment for TB last time?	Govt. hospitals/chest hospitals<br>Govt. TB clinics<br>Upazila health complex<br>BRAC DOTS centers<br>Nagar shaystho kendro<br>Surjeyer hasi<br>Blue star(SMC)<br>TB screening and treatment center, icddr,b<br>Others (please specify___)	1<br>2<br>3<br>4<br>5<br>6<br>7<br>8<br>9		

Sl#	Questions	Types of code		Direction	Comments
		Don't know<br>No response	97<br>98		

Section 14: Violence and Sexual harassment
(Now, I will ask you about your violence and sexual harassment)

Q. No.	Questions	Coding categories	Skip to	Comments
1401	In the last 12 months, were you ever beaten?	Yes 1<br>No 2<br>Don't remember 97<br>No response 98	No/Don't remember/No response → **1403**	
1402	In the last 12 months, who had beaten you?<br><br>**(Multiple answers possible)**<br>**(DO NOT READ OUT)**<br>**Circle 1 if mentioned**<br>**Circle 2 if not mentioned**	Men in uniform 1 2<br>Mastans 1 2<br>New Client 1 2<br>Regular client 1 2<br>Local people 1 2<br>Family members/Relatives 1 2<br>(Relation)		
1403	In the last 12 months, were you forced to have sex with someone even though you did not want to?	Yes 1<br>No 2<br>Don't know/Can't remember 97<br>No response 98	No/Don't know/No response → **End**	
1404	Last year who was the person (or people) who physically forced you to have sex against your will?<br><br>(Multiple answers possible)<br>(DO NOT READ OUT)<br>Circle 1 if mentioned<br>Circle 2 if not mentioned	Men in uniform 1 2<br>Mastans 1 2<br>New Client 1 2<br>Regular client 1 2<br>Local people 1 2<br>Family members/Relatives 1 2<br>(Relation)		

Thank you very much for your kind cooperation and spending your valuable time with me.

Annex 6: Post-test Counselling form for female sex partner of HIV positive male MWID (English)

Unique ID ☐☐☐

1. Result provided: *(Please circle code)*

☐ 1. HIV antibody negative ☐ 2. HIV antibody positive ☐ 3. Indeterminate

2.USE ONLY FOR HIV NEGATIVE RESULT PROVISION **(CERTIFICATION FROM COUNSELLOR DURING THIS SESSION):**

- ☐ Provided & explained client result
- ☐ Checked for window period and subsequent exposure
- ☐ Client advised to re-test: 1. Yes 2. No
- ☐ Provision of risk reduction counselling

3. INDETERMINATE RESULT ONLY:

- ☐ Explained the possibility that testing has been performed during the window period.
- ☐ Avoid unprotected intercourse or sharing injecting equipment
- ☐ Re testing at this centre in 12 weeks (4 to 6 weeks in case of pregnancy, when applicable)
- ☐ Stress management and supportive counselling

4. Use only for HIV positive result provision:

4.1 Certification from counsellor during post-test counselling

- ☐ Assessed readiness of client for result
- ☐ Assessed suicidal risk of the client
- ☐ Assessed whether the client requires assistance from family members
- ☐ Assessed whether the client requires ongoing counselling support
- ☐ Assessed whether the client requires financial assistance
- ☐ Assessed whether the client requires treatment assistance
- ☐ Assessed whether the client requires treatment for mental health
- ☐ Provided brief information about follow-up, treatment, care and support network
- ☐ Discussion on strategies for partner disclosure
- ☐ Ensured that the client can get back home safely
- ☐ Others (specify): …………………………………………………

4.2: Whether the client is referred to implementing partners for care, support and treatment (CST) services:

1. Yes 2. No If yes, where (name of organization)? ---

Reason for referral: ---

Other comments (if any): ---

Name of Counsellor Name **Signature of Counsellor** **Date**
