# Supplementary material for: Prevalence of HIV, risk behaviours and vulnerabilities of female sex partners of the HIV positive people who inject drugs (PWID) in Dhaka city, Bangladesh
Source: PLoS One. 2023 Jun 5;18(6):e0286673. doi: 10.1371/journal.pone.0286673 (PMC10241362; doi:10.1371/journal.pone.0286673)
Supplement: S2 File — (PDF) [file pone.0286673.s002.pdf]

icddr,b

Annex 1: Consent Form for HIV positive men who inject drugs (MWID) (Bengali)

একক আইডি ঃ | | | | | | এমএমটি | | | | | PWID | | | | |

Protocol No. : **PR-19019**

গবেষণার উদ্দেশ্য:

আমি আইসিডিডিআর,বি (কলেরা হাসপাতাল, মহাখালী, ঢাকা) থেকে এসেছি। আমার নাম------------------------। আমি জানি যে, আপনি এইচআইভি পজিটিভ এবং সেভ দা সিলড্রেন/কেয়ার বাংলাদেশ কর্তৃক পরিচালিত ড্রপ-ইন-সেন্টার (ডিআইসি) থেকে এইচআইভি প্রতিরোধ মূলক সেবা পেয়ে আসছেন। আপনি হয়ত অবগত আছেন যে, ইঞ্জেকশানের মাধ্যমে মাদকগ্রহনকারী (পিডব্লিউআইডি) এইচআইভি পজিটিভ এর মহিলা যৌন সঙ্গী (স্ত্রী, মহিলা যৌন কর্মী এবং অন্যান্য মহিলা যৌন সঙ্গী) হিসেবে আপনারা এইচআইভি সংক্রমনের জন্য ঝুঁকিপুর্ণ। অতএব এটা জানা দরকার যে আপনি যদি এইচআইভি সংক্রমনে আক্রান্ত হোন তাহলে বাংলাদেশ সরকারের কাছ থেকে বিনামুল্যে চিকিৎসা ব্যবস্থা নিশ্চিত করা হবে এবং গুরুতর রোগের প্রতিরোধের ব্যবস্থা নেওয়া হবে।

গবেষণা পদ্ধতি:

এই গবেষণায়, ওরাকুইক এর মাধ্যমে মুখের ভিতর থেকে লালা ব্যবহার করে এইচআইভি পরীক্ষা করা হবে যা করতে প্রায় আধা ঘন্টা সময় নিতে পারে। যদি এই ওরাকুইক পরীক্ষায় কাউকে পজিটিভ পাওয়া যায় তাহলে আইসিডিডিআর,বিতে, ডব্লিউএইচও তিনটি টেষ্টের মাধম্যে এইচআইভি পরীক্ষা করা হবে যা করতে ৫মি.লি. রক্ত (একটি পূর্ণ চা চামচ) পরীক্ষার জন্য নেওয়া হবে। ওরাকুইক এর মাধ্যমে এইচআইভি পরীক্ষার পর, আর্থ-সামাজিক, ইঞ্জেকশান্ এবং যৌন ঝুঁকির আচরণ, এইচআইভির দূর্বলতা এবং এইচআইভির প্রতিরোধের সেবা সমন্ধে তথ্য সংগ্রহের জন্য আরও ৩০-৪০ মিনিট সময় নেওয়া হবে। পরীক্ষা এবং সাক্ষাৎকারটি গোপনীয়তা বজায় রেখে বাসস্থান বা কোন উপযুক্ত জায়গা বা ডিআইসিতে যেখানে উত্তরদাতা আরামদায়ক বোধ করবে সেখানে নেয়া হবে। যদি মায়ের এইচআইভি পজিটিভ হয় এবং আপনি ও আপনার স্ত্রী/মহিলা যৌন সঙ্গী রাজী থাকেন তাহলে ১৭ বছর পর্যন্ত বাচ্চাদেরও পরীক্ষা করা হবে। দুই বছরের কম বয়সী শিশুদের, ৫মিঃলিঃ রক্ত নিয়ে ভাইরাল নিউক্লিয়িক এসিড এবং ২-১৭ বছর বয়সের শিশুদের জন্য ওরাকুইক এইচআইভি পরীক্ষা করার জন্য ব্যবহার করা হবে। এইচআইভি পরীক্ষার পূর্বে, একজন মহিলা কাউন্সেলর তার কাছ থেকে লিখিত সম্মতি গ্রহণ করবেন। পরীক্ষার পর যদি নেগিটিভ পাওয়া যায় তাহলে, তাকে স্পটে ফলাফল জানিয়ে দেওয়া হবে এবং তাকে সেখানে পরীক্ষা পরবর্তী কাউন্সেলিং করা হবে। এইচআইভি পরীক্ষা নি:শ্চিন্ত করার (যদি প্রয়োজন হয়) পর তার পরীক্ষা পরবর্তী কাউন্সেলিং করা হবে এবং যদি পজিটিভ পাওয়া যায়, তাহলে তাকে বিনামূল্যে সেবা বাস্তবায়নকারী অংশীদারদের কাছে চিকিৎসা, যত্ন এবং সহায়তার সেবা দেওয়ার জন্য পাঠানো হবে।

আপনার কাছ থেকে কি আশা করা হয়?

ঢাকা শহরের পিডব্লিউআইডি-এর মধ্যে এইচআইভির বর্তমান অবস্থা বিবেচনা করে পুরুষ এইচআইভি পজিটিভ পিডব্লিউআইডি এর সকল মহিলা যৌন সঙ্গীদের মধ্যে এইচআইভি পরীক্ষা জরুরিভাবে প্রয়োজন। অতএব, আমরা আশা করছি যে আপনি লিখিত সম্মতি পূরণ করে অনুগ্রহপূর্বক অনুমতি দেবেন যাতে আমরা এই গবেষণায় আপনার মহিলা যৌন সঙ্গী (পত্নী /এফএসডব্লিউ/ অন্য মহিলা যৌন সঙ্গী) নিতে পারি। যদি আপনার পত্নী HIV পজিটিভ হয় তবে আমরা আপনার সন্তান যদি ১৮ বছরের নীচে থাকে তাহলে তাদেরও আমরা এই গবেষনায় অন্তর্ভুক্ত করবো।

ঝুঁকি এবং সুবিধা

ওরাকুইকের সময় যদি এইচআইভি পজিটিভ পাওয়া যায় তবে আমাদের ৫ মিলিলিটার রক্ত নিতে হবে এবং সকল জীবাণুমুক্ত সতর্কতা নেওয়া হবে। এটি একটি নির্জীব প্রক্রিয়া এবং রক্ত সংগ্রহের সময় সামান্য অস্বস্তি হতে পারে। যদি আপনার স্ত্রী/ ট্রানজেকশনাল মহিলা যৌন সঙ্গী/ নন ট্রানজেকশনাল মহিলা যৌন সঙ্গী এইচআইভি পজিটিভ হয়, তবে সরকারী বিধি মোতাবেক আপনাদের দুজনকেই বিনামুল্যে চিকিৎসা নি:শ্চিতকরার জন্য আপনার এইচআইভি ফলাফল আপনার মহিলা যৌন সঙ্গীকে জানানোর প্রয়োজন হবে। আমরা আশা করি এই গবেষণায় প্রাপ্ত তথ্যটি এইচআইভি পরীক্ষা গ্রহণযোগ্যতা বাড়িয়ে তুলতে সহায়ক হবে যা এইচআইভি পজিটিভদের জন্য চিকিৎসা করা সম্ভব হবে। পুরুষ এইচআইভি পজিটিভ পিডব্লিউআইডি এর মহিলা যৌন সঙ্গীরা পরোক্ষভাবে উপকার পাবেন যা এই গবেষণার থেকে প্রাপ্ত ফলাফল জাতীয় পর্যায়ে এইচআইভি সংক্রান্ত নীতি ও প্রতিরোধ কার্যক্রম পরিচালনা করতে ব্যবহৃত হবে।

ব্যক্তিগত, নামবিহীন এবং গোপনীয়তা

অংশগ্রহণ এবং আপনার মহিলা যৌন সঙ্গীর দেয়া তথ্য সমুহ অত্যন্ত গোপনীয়ভাবে সংরক্ষণ করা হবে। ওরাকুইক ব্যবহার করে এইচআইভি পরীক্ষা এবং ঝুঁকি আচরণ সাক্ষাৎকার বাড়ীতে গোপনীয় জায়গায় বা ডিআইসি বা একটি উপযুক্ত স্থানে সম্পন্ন করা হবে যেখানে আপনি আরামদায়ক মনে করেন।

তথ্য ভবিষ্যতে ব্যবহার

যদি পর্যাপ্ত তহবিলের ব্যবস্থা করা যায় তাহলে হেপাটাইটিস সি ভাইরাসের সনাক্তকরণ এবং এইচআইভি ঔষধের প্রতিরোধের উপর আরও পরীক্ষা করার জন্য আমরা ভবিষ্যতে সম্ভাব্য ব্যবহারের জন্য ৫ বছরের জন্য পুরো রক্ত এবং সিরামের নমুনা সংরক্ষণ করব। নমুনাগুলি আইসিডিডিআর,বি এর ভাইরোলজী গবেষণাগারে সংরক্ষণ করা হবে এবং একই গবেষণাগারের তত্ত্বাবধানে থাকবে। এই সমস্ত সঞ্চিত নমুনাগুলিতে কেবল বয়স, লিঙ্গ, গবেষণা নাম এবং দৈবচয়িত আইডি নম্বর থাকবে; অন্যান্য কোন তথ্য লেবেলে রেকর্ড করা হবে না।

অংশগ্রহণ না করা এবং প্রত্যাহার এর অধিকার

এই গবেষণায় আপনার অংশগ্রহণ একান্তই স্বেচ্ছামূলক। তিনি যে কোনও বা সমস্ত প্রশ্নের উত্তর না দিতে এবং লালা বা রক্তের নমুনা সরবরাহ না করতে পারবেন। তিনি যে কোনো সময় এমনকি একটি সাক্ষাৎকার এর মধ্যবর্তী সময়ে গবেষণা ছেড়ে যেতে পারবেন। আপনি এই গবেষণায় অংশগ্রহণ প্রত্যাখ্যান করার অধিকার রাখেন, এসব ক্ষেত্রে যা আপনার জন্য বিদ্যমান এইচআইভি প্রতিরোধ সেবা এবং চিকিৎসা সুবিধা গ্রহণে বাধাগ্রস্থ করবে না।

ক্ষতিপূরণ বিষয়ক নীতিমালা

এইচআইভি পজিটিভ ব্যক্তিদের জন্য চিকিৎসা একেবারে বিনামূল্যে। যদি আপনাকে প্রয়োজন হয়, তাহলে এই গবেষণার উদ্দেশ্যে ডিআইসি-তে আসার জন্য সঙ্গীসহ যাতায়াত ভাতা দেয়া হবে। উত্তরদাতাকে আমাদের কাছে নিয়ে আসার জন্য আপনাকে যাতায়াত ভাতা দেয়া হবে। সাক্ষাৎকার শেষে উত্তরদাতাকে কিছু নাস্তা দেয়া হবে।

ব্যাক্তিগত যোগাযোগ

আপনার কোন প্রশ্ন থাকলে অনুগ্রহ করে নিঃসংকোচে জিজ্ঞাসা করুন। আপনার যদি পরেও কোন প্রশ্ন জাগে সেক্ষেত্রে আপনার প্রশ্নের উত্তরের জন্য আপনি এই গবেষণার প্রধান গবেষক মোঃ মাসুদ রেজা, আইসিডিডিআর,বি, মহাখালী, ঢাকা, ফোনঃ ৯৮২৭০০১-১০, এক্সটেনশান-৪২০৫ তে যোগাযোগ করুন।

আপনি আপনার মহিলা যৌন সঙ্গী/সন্তান সহ যদি আমাদের গবেষণায় অর্ন্তভুক্ত হতে সম্মত থাকেন, তাহলে নিচের নির্দিষ্ট স্থানে আপনার স্বাক্ষর বা বাম বৃদ্ধাঙ্গুলীর টিপসই দিন।

আপনার সহযোগীতার জন্য অশেষ ধন্যবাদ।

____ ____________

এইচআইভি পজিটিভ পিডব্লিউআইডি এর স্বাক্ষর বা বাম বৃদ্ধাঙ্গুলীর টিপসই তারিখ

______________________________ ____________

প্রধান গবেষক বা তার প্রতিনিধির স্বাক্ষর তারিখ

যদি রাজি না থাকে বিস্তাড়িত লিখুন:

Annex 2: Consent Form for female sex partner of HIV positive MWID (Bengali)

(For those who are 18 years of above)

জনসংখ্যা গোষ্ঠী	কোড (বৃত্ত আঁকুন যেখানে প্রযোজ্য)
স্ত্রী	১
মহিলা যৌন কর্মী	২
বিবাহিত পিডব্লিউআইডি এর অন্যান্য মহিলা যৌন সঙ্গী (স্ত্রী এবং মহিলা যৌন কর্মী ব্যতীত)	৩
অবিবাহিত পিডব্লিউআইডি এর অন্যান্য মহিলা যৌন সঙ্গী (স্ত্রী এবং মহিলা যৌন কর্মী ব্যতীত)	৪

একক আইডি :

Protocol No. : PR-19019

গবেষণার উদ্দেশ্য:

আমি আইসিডিডিআর,বি (কলেরা হাসপাতাল, মহাখালী, ঢাকা) থেকে এসেছি। আমার নাম------------------------। আপনি হয়ত অবগত আছেন যে, ইঞ্জেকশানের মাধ্যমে মাদকগ্রহনকারী (পিডব্লিউআইডি) এইচআইভি পজিটিভ এর মহিলা যৌন সঙ্গী (স্ত্রী, মহিলা যৌন কর্মী এবং অন্যান্য মহিলা যৌন সঙ্গী) এবং সন্তান হিসেবে আপনারা এইচআইভি সংক্রমনের জন্য ঝুঁকিপুর্ণ। অতএব এটা জানা দরকার যে আপনি যদি এইচআইভি সংক্রমনে আক্রান্ত হোন তাহলে বাংলাদেশ সরকারের কাছ থেকে বিনামুল্যে চিকিৎসা ব্যবস্থা নিশ্চিত করা হবে এবং গুরুতর রোগের প্রতিরোধের ব্যবস্থা নেওয়া হবে।

গবেষনা পদ্বতি:

এই গবেষণায়, ওরাকুইক এর মাধ্যমে মুখের ভিতর থেকে লালা ব্যবহার করে এইচআইভি পরীক্ষা করা হবে যা করতে প্রায় আধা ঘন্টা সময় নিতে পারে। যদি এই ওরাকুইক পরীক্ষায় কাউকে পজিটিভ পাওয়া যায় তাহলে আইসিডিডিআরবি,তে, ডব্লিউএইচও তিনটি টেষ্টের মাধম্যে এইচআইভি পরীক্ষা করা হবে যা করতে ৫মি.লি. রক্ত (একটি পূর্ণ চা চামচ) পরীক্ষার জন্য নেওয়া হবে। ওরাকুইক এর মাধ্যমে এইচআইভি পরীক্ষার পর, আর্থ-সামাজিক, ইঞ্জেকশান্ এবং যৌন ঝুঁকির আচরণ, এইচআইভির দূর্বলতা এবং এনজিও হতে এইচআইভির প্রতিরোধের সেবা সমন্ধে তথ্য সংগ্রহের জন্য আরও ৩০-৪০ মিনিট সময় নেওয়া হবে। পরীক্ষা এবং সাক্ষাৎকারটি গোপনীয়তা বজায় রেখে বাসস্থান বা কোন উপযুক্ত জায়গা বা ডিআইসিতে যেখানে উত্তরদাতা আরামদায়ক বোধ করবে সেখানে নেয়া হবে। যদি আপনি এবং আপনার স্ত্রী/মহিলা যৌন সঙ্গী রাজী থাকেন তাহলে ১৭ বছর পর্যন্ত বাচ্চাদেরও অর্ন্তভূক্ত করা হবে। দুই বছরের কম বয়সী শিশুদের, ৫মিঃলিঃ (একটি পূর্ণ চা চামচ) রক্ত নিয়ে ভাইরাল নিউক্লিয়িক এসিড এবং ২-১৭ বছর বয়সের শিশুদের জন্য ওরাকুইক এইচআইভি পরীক্ষা করার জন্য ব্যবহার করা হবে।

এইচআইভি পরীক্ষার পূর্বে, একজন মহিলা কাউন্সেলর তার কাছ থেকে লিখিত সম্মতি গ্রহণ করবেন। পরীক্ষার পর যদি নেগিটিভ পাওয়া যায় তাহলে, তাকে স্পটে ফলাফল জানিয়ে দেওয়া হবে এবং তাকে সেখানে পরীক্ষা পরবর্তী কাউন্সেলিং করা হবে, সেখানে ঝুঁকি আচরণের সাক্ষাৎকার এ এইচআইভি পরীক্ষা নিশ্চত করার (যদি প্রয়োজন হয়) পর তার পরীক্ষা পরবর্তী কাউন্সেলিং করা হবে এবং যদি পজিটিভ পাওয়া যায়, তাহলে তাকে বিনামূল্যে সেবা বাস্তবায়নকারী অংশীদারদের কাছে চিকিৎসা, যত্ন এবং সহায়তার সেবা দেওয়ার জন্য পাঠানো হবে।

আপনার কাছ থেকে কি আশা করা হয়?

ঢাকা শহরের পিডব্লিউআইডি-এর মধ্যে এইচআইভির বর্তমান অবস্থা বিবেচনা করে পুরুষ এইচআইভি পজিটিভ পিডব্লিউআইডি এর সকল মহিলা যৌন সঙ্গীদের এবং তাদের ১৭বছর বয়স পর্যন্ত বাচ্চাদের মধ্যে এইচআইভি পরীক্ষা জরুরিভাবে প্রয়োজন। অতএব, আমরা আশা করছি যে আপনি অনুগ্রহপূর্বক অনুমতি দেবেন এই গবেষণায় অংশ নিতে।

ঝুঁকি এবং সুবিধা

ওরাকুইকের সময় যদি এইচআইভি পজিটিভ পাওয়া যায় তবে আমাদের ৫ মিলিলিটার রক্ত নিতে হবে এবং সব সকল জীবাণুমুক্ত সতর্কতা নেওয়া হবে। এটি একটি নির্জীব প্রক্রিয়া এবং রক্ত সংগ্রহের সময় সামান্য অস্বস্তি হতে পারে। যদি আপনার এইচআইভি পরীক্ষার ফলাফল পজিটিভ হয়, তবে সরকারী বিধি মোতাবেক বিনামুল্যে চিকিৎসা নি:শ্চিতকরার জন্য পরীক্ষার ফলাফল আপনার স্বামী / আপনার অভিভাবককে জানানোর প্রয়োজন হবে। আমরা আশা করি এই গবেষণায় প্রাপ্ত তথ্যটি এইচআইভি পরীক্ষা গ্রহণযোগ্যতা বাড়িয়ে তুলতে সহায়ক হবে যা এইচআইভি পজিটিভদের জন্য চিকিৎসা করা সম্ভব হবে। পুরুষ এইচআইভি পজিটিভ পিডব্লিউআইডি এর মহিলা যৌন সঙ্গীরা পরোক্ষভাবে উপকার পাবেন যা এই গবেষণার থেকে প্রাপ্ত ফলাফল জাতীয় পর্যায়ে এইচআইভি সংক্রান্ত নীতি ও প্রতিরোধ কার্যক্রম পরিচালনা করতে ব্যবহৃত হবে।

ব্যক্তিগত, নামবিহীন এবং গোপনীয়তা

অংশগ্রহণ এবং আপনার দেয়া তথ্য সমুহ অত্যন্ত গোপনীয়ভাবে সংরক্ষণ করা হবে। ওরাকুইক ব্যবহার করে এইচআইভি পরীক্ষা এবং ঝুঁকি আচরণ সাক্ষাৎকার বাড়ীতে গোপনীয় জায়গায় বা ডিআইসি বা একটি উপযুক্ত স্থানে সম্পন্ন করা হবে যেখানে আপনি আরামদায়ক মনে করেন।

তথ্য ভবিষ্যতে ব্যবহার

যদি পর্যাপ্ত তহবিলের ব্যবস্থা করা যায় তাহলে হেপাটাইটিস সি এবং এইচআইভি ঔষধের প্রতিরোধের উপর আরও পরীক্ষা করার জন্য আমরা ভবিষ্যতে সম্ভাব্য ব্যবহারের জন্য ৫ বছরের জন্য পুরো রক্ত এবং সিরামের নমুনা সংরক্ষণ করব। নমুনাগুলি আইসিডিডিআর,বি এর ভাইরোলজী গবেষণাগারে সংরক্ষণ করা হবে এবং একই গবেষণাগারের তত্ত্বাবধানে থাকবে। এই সমস্ত সঞ্চিত নমুনাগুলিতে কেবল বয়স, লিঙ্গ, গবেষণা নাম এবং দৈবচয়িত আইডি নম্বর থাকবে; অন্যান্য কোন তথ্য লেবেলে রেকর্ড করা হবে না।

অংশগ্রহণ না করা এবং প্রত্যাহার এর অধিকার

এই গবেষণায় আপনার অংশগ্রহণ একান্তই স্বেচ্ছামূলক। আপনি যে কোনও বা সমস্ত প্রশ্নের উত্তর না দিতে এবং লালা বা রক্তের নমুনা সরবরাহ না করতে পারবেন। আপনি যে কোনো সময় এমনকি একটি সাক্ষাৎকার এর মধ্যবর্তী সময়ে গবেষণা ছেড়ে যেতে পারবেন। আপনি এই গবেষণায় অংশগ্রহণ প্রত্যাখ্যান করার অধিকার রাখেন, এসব ক্ষেত্রে যা আপনার স্বামী/ছেলে বন্ধু/যৌন সঙ্গীর জন্য বিদ্যমান ডিআইসি ও আউটরীচ এ এইচআইভি প্রতিরোধ সেবা এবং চিকিৎসা সুবিধা গ্রহণে বাধাগ্রস্থ করবে না।

ক্ষতিপূরণ বিষয়ক নীতিমালা

এইচআইভি পজিটিভ ব্যক্তিদের জন্য চিকিৎসা একেবারে বিনামূল্যে। যদি আপনাকে প্রয়োজন হয়, তাহলে এই গবেষণার উদ্দেশ্যে ডিআইসি-তে আসার জন্য স্বামী/সঙ্গীসহ যাতায়াত ভাতা দেয়া হবে। সাক্ষাৎকার শেষে উত্তরদাতাকে কিছু নাস্তা দেয়া হবে।

ব্যাক্তিগত যোগাযোগ

আপনার কোন প্রশ্ন থাকলে অনুগ্রহ করে নিঃসংকোচে জিজ্ঞাসা করুন। আপনার যদি পরেও কোন প্রশ্ন জাগে সেক্ষেত্রে আপনার প্রশ্নের উত্তরের জন্য আপনি এই গবেষণার প্রধান গবেষক মোঃ মাসুদ রেজা, আইসিডিডিআর,বি, মহাখালী, ঢাকা, ফোনঃ ৯৮২৭০০১-১০, এক্সটেনশান-৪২০৫ তে যোগাযোগ করুন।

আপনি যদি আমাদের গবেষণায় অর্ন্তভুক্ত হতে সম্মত থাকেন বা আপনার ১৭ বছর বয়স পর্যন্ত সন্তানকে আমাদের গবেষণায় অর্ন্তভুক্ত হতে সম্মত হন তাহলে নিচের নির্দিষ্ট স্থানে আপনার স্বাক্ষর বা বাম বৃদ্ধাঙ্গুলীর টিপসই দিন।

আপনার সহযোগীতার জন্য অশেষ ধন্যবাদ।

______________________________________ ____________

উত্তরদাতার স্বাক্ষর বা বাম বৃদ্ধাঙ্গুলীর টিপসই তারিখ

________________________ ____________

প্রধান গবেষক বা তার প্রতিনিধির স্বাক্ষর তারিখ

যদি রাজি না থাকে বিস্তারিত লিখুন:

Annex 3: Assent Form for female sex partner of HIV positive MWID (Bengali)

(For those who are 15 to less than 18 years)

জনসংখ্যা গোষ্ঠী	কোড (বৃত্ত আঁকুন যেখানে প্রযোজ্য)
স্ত্রী	১
মহিলা যৌন কর্মী	২
বিবাহিত পিডব্লিউআইডি এর অন্যান্য মহিলা যৌন সঙ্গী (স্ত্রী এবং মহিলা যৌন কর্মী ব্যতীত)	৩
অবিবাহিত পিডব্লিউআইডি এর অন্যান্য মহিলা যৌন সঙ্গী (স্ত্রী এবং মহিলা যৌন কর্মী ব্যতীত)	৪

একক আইডি :

Protocol No. : PR-19019

গবেষণার উদ্দেশ্য:

আমি আইসিডিডিআর,বি (কলেরা হাসপাতাল, মহাখালী, ঢাকা) থেকে এসেছি। আমার নাম-------------------------। আপনি হয়ত অবগত আছেন যে, ইঞ্জেকশানের মাধ্যমে মাদকগ্রহনকারী (পিডব্লিউআইডি) এইচআইভি পজিটিভ এর মহিলা যৌন সঙ্গীঁ (স্ত্রী, মহিলা যৌন কর্মী এবং অন্যান্য মহিলা যৌন সঙ্গীঁ) এবং সন্তান হিসেবে আপনারা এইচআইভি সংক্রমনের জন্য ঝুঁকিপুর্ণ। অতএব এটা জানা দরকার যে আপনি যদি এইচআইভি সংক্রমনে আক্রান্ত হোন তাহলে বাংলাদেশ সরকারের কাছ থেকে বিনামুল্যে চিকিৎসা ব্যবস্থা নিশ্চিত করা হবে এবং গুরুতর রোগের প্রতিরোধের ব্যবস্থা নেওয়া হবে।

গবেষনা পদ্বতি:

এই গবেষণায়, ওরাকুইক এর মাধ্যমে মূখের ভিতর থেকে লালা ব্যবহার করে এইচআইভি পরীক্ষা করা হবে যা করতে প্রায় আধা ঘন্টা সময় নিতে পারে। যদি এই ওরাকুইক পরীক্ষায় কাউকে পজিটিভ পাওয়া যায় তাহলে আইসিডিডিআরবি,তে, ডব্লিউএইচও তিনটি টেষ্টের মাধম্যে এইচআইভি পরীক্ষা করা হবে যা করতে ৫মি.লি. রক্ত (একটি পূর্ণ চা চামচ) পরীক্ষার জন্য নেওয়া হবে। ওরাকুইক এর মাধ্যমে এইচআইভি পরীক্ষার পর, আর্থ-সামাজিক, ইঞ্জেকশান্ এবং যৌন ঝুঁকির আচরণ, এইচআইভির দূর্বলতা এবং এনজিও হতে এইচআইভির প্রতিরোধের সেবা সমন্ধে তথ্য সংগ্রহের জন্য আরও ৩০-৪০ মিনিট সময় নেওয়া হবে। পরীক্ষা এবং সাক্ষাৎকারটি গোপনীয়তা বজায় রেখে বাসস্থান বা কোন উপযুক্ত জায়গা বা ডিআইসিতে যেখানে উত্তরদাতা আরামদায়ক বোধ করবে সেখানে নেয়া হবে। যদি আপনি এবং আপনার স্ত্রী/মহিলা যৌন সঙ্গী রাজী থাকেন তাহলে ১৭ বছর পর্যন্ত বাচ্চাদেরও অর্ন্তভূক্ত করা হবে। দুই বছরের কম বয়সী শিশুদের, ৫মিঃলিঃ (একটি পূর্ণ চা চামচ) রক্ত নিয়ে ভাইরাল নিউক্লিয়িক এসিড এবং ২-১৭ বছর বয়সের শিশুদের জন্য ওরাকুইক এইচআইভি পরীক্ষা করার জন্য ব্যবহার করা হবে।

এইচআইভি পরীক্ষার পূর্বে, একজন মহিলা কাউন্সেলর তার কাছ থেকে লিখিত সম্মতি গ্রহণ করবেন। পরীক্ষার পর যদি নেগিটিভ পাওয়া যায় তাহলে, তাকে স্পটে ফলাফল জানিয়ে দেওয়া হবে এবং তাকে সেখানে পরীক্ষা পরবর্তী কাউন্সেলিং করা হবে, সেখানে ঝুঁকি আচরণের সাক্ষাৎকার এ এইচআইভি পরীক্ষা নিশ্চত করার (যদি প্রয়োজন হয়) পর তার পরীক্ষা পরবর্তী কাউন্সেলিং করা হবে এবং যদি পজিটিভ পাওয়া যায়, তাহলে তাকে বিনামূল্যে সেবা বাস্তবায়নকারী অংশীদারদের কাছে চিকিৎসা, যত্ন এবং সহায়তার সেবা দেওয়ার জন্য পাঠানো হবে।

আপনার কাছ থেকে কি আশা করা হয়?

ঢাকা শহরের পিডব্লিউআইডি-এর মধ্যে এইচআইভির বর্তমান অবস্থা বিবেচনা করে পুরুষ এইচআইভি পজিটিভ পিডব্লিউআইডি এর সকল মহিলা যৌন সঙ্গীদের এবং তাদের ১৭বছর বয়স পর্যন্ত বাচ্চাদের মধ্যে এইচআইভি পরীক্ষা জরুরিভাবে প্রয়োজন। অতএব, আমরা আশা করছি যে আপনি অনুগ্রহপূর্বক অনুমতি দেবেন এই গবেষণায় অংশ নিতে।

ঝুঁকি এবং সুবিধা

ওরাকুইকের সময় যদি এইচআইভি পজিটিভ পাওয়া যায় তবে আমাদের ৫ মিলিলিটার রক্ত নিতে হবে এবং সব সকল জীবাণুমুক্ত সতর্কতা নেওয়া হবে। এটি একটি নির্জীব প্রক্রিয়া এবং রক্ত সংগ্রহের সময় সামান্য অস্বস্তি হতে পারে। যদি আপনার এইচআইভি পরীক্ষার ফলাফল পজিটিভ হয়, তবে সরকারী বিধি মোতাবেক বিনামুল্যে চিকিৎসা নি:শ্চিতকরার জন্য পরীক্ষার ফলাফল আপনার স্বামী / আপনার অভিভাবককে জানানোর প্রয়োজন হবে। আমরা আশা করি এই গবেষণায় প্রাপ্ত তথ্যটি এইচআইভি পরীক্ষা গ্রহণযোগ্যতা বাড়িয়ে তুলতে সহায়ক হবে যা এইচআইভি পজিটিভদের জন্য চিকিৎসা করা সম্ভব হবে। পুরুষ এইচআইভি পজিটিভ পিডব্লিউআইডি এর মহিলা যৌন সঙ্গীরা পরোক্ষভাবে উপকার পাবেন যা এই গবেষণার থেকে প্রাপ্ত ফলাফল জাতীয় পর্যায়ে এইচআইভি সংক্রান্ত নীতি ও প্রতিরোধ কার্যক্রম পরিচালনা করতে ব্যবহৃত হবে।

ব্যক্তিগত, নামবিহীন এবং গোপনীয়তা

অংশগ্রহণ এবং আপনার দেয়া তথ্য সমুহ অত্যন্ত গোপনীয়ভাবে সংরক্ষণ করা হবে। ওরাকুইক ব্যবহার করে এইচআইভি পরীক্ষা এবং ঝুঁকি আচরণ সাক্ষাৎকার বাড়ীতে গোপনীয় জায়গায় বা ডিআইসি বা একটি উপযুক্ত স্থানে সম্পন্ন করা হবে যেখানে আপনি আরামদায়ক মনে করেন।

তথ্য ভবিষ্যতে ব্যবহার

যদি পর্যাপ্ত তহবিলের ব্যবস্থা করা যায় তাহলে হেপাটাইটিস সি এবং এইচআইভি ঔষধের প্রতিরোধের উপর আরও পরীক্ষা করার জন্য আমরা ভবিষ্যতে সম্ভাব্য ব্যবহারের জন্য ৫ বছরের জন্য পুরো রক্ত এবং সিরামের নমুনা সংরক্ষণ করব। নমুনাগুলি আইসিডিডিআর,বি এর ভাইরোলজী গবেষণাগারে সংরক্ষণ করা হবে এবং একই গবেষণাগারের তত্ত্বাবধানে থাকবে। এই সমস্ত সঞ্চিত নমুনাগুলিতে কেবল বয়স, লিঙ্গ, গবেষণা নাম এবং দৈবচয়িত আইডি নম্বর থাকবে; অন্যান্য কোন তথ্য লেবেলে রেকর্ড করা হবে না।

অংশগ্রহণ না করা এবং প্রত্যাহার এর অধিকার

এই গবেষণায় আপনার অংশগ্রহণ একান্তই স্বেচ্ছামূলক। আপনি যে কোনও বা সমস্ত প্রশ্নের উত্তর না দিতে এবং লালা বা রক্তের নমুনা সরবরাহ না করতে পারবেন। আপনি যে কোনো সময় এমনকি একটি সাক্ষাৎকার এর মধ্যবর্তী সময়ে গবেষণা ছেড়ে যেতে পারবেন। আপনি এই গবেষণায় অংশগ্রহণ প্রত্যাখ্যান করার অধিকার রাখেন, এসব ক্ষেত্রে যা আপনার স্বামী/ছেলে বন্ধু/যৌন সঙ্গীর জন্য বিদ্যমান ডিআইসি ও আউটরীচ এ এইচআইভি প্রতিরোধ সেবা এবং চিকিৎসা সুবিধা গ্রহণে বাধাগ্রস্থ করবে না।

ক্ষতিপূরণ বিষয়ক নীতিমালা

এইচআইভি পজিটিভ ব্যক্তিদের জন্য চিকিৎসা একেবারে বিনামূল্যে। যদি আপনাকে প্রয়োজন হয়, তাহলে এই গবেষণার উদ্দেশ্যে ডিআইসি-তে আসার জন্য স্বামী/সঙ্গীসহ যাতায়াত ভাতা দেয়া হবে। সাক্ষাৎকার শেষে উত্তরদাতাকে কিছু নাস্তা দেয়া হবে।

ব্যাক্তিগত যোগাযোগ

আপনার কোন প্রশ্ন থাকলে অনুগ্রহ করে নিঃসংকোচে জিজ্ঞাসা করুন। আপনার যদি পরেও কোন প্রশ্ন জাগে সেক্ষেত্রে আপনার প্রশ্নের উত্তরের জন্য আপনি এই গবেষণার প্রধান গবেষক মোঃ মাসুদ রেজা, আইসিডিডিআর,বি, মহাখালী, ঢাকা, ফোনঃ ৯৮২৭০০১-১০, এক্সটেনশান-৪২০৫ তে যোগাযোগ করুন।

আপনি যদি আমাদের গবেষণায় অর্ন্তভুক্ত হতে সম্মত থাকেন বা আপনার ১৭ বছর বয়স পর্যন্ত সন্তানকে আমাদের গবেষণায় অর্ন্তভুক্ত হতে সম্মত হন তাহলে নিচের নির্দিষ্ট স্থানে আপনার স্বাক্ষর বা বাম বৃদ্ধাঙ্গুলীর টিপসই দিন।

আপনার সহযোগীতার জন্য অশেষ ধন্যবাদ।

____ ____________

উত্তরদাতার স্বাক্ষর বা বাম বৃদ্ধাঙ্গুলীর টিপসই তারিখ

অভিভাবকের সম্মতি

আপনি কি মহিলাকে এই গবেষণায় অংশগ্রহণ করার অনুমতি দিতে যদি সম্মত থাকেন, তাহলে নিচের নির্দিষ্ট স্থানে আপনার স্বাক্ষর বা বাম বৃদ্ধাঙ্গুলীর টিপসই দিন।

____ ____________

অভিভাবক এর স্বাক্ষর বা বাম বৃদ্ধাঙ্গুলীর টিপসই তারিখ

______________________________ ____________

প্রধান গবেষক বা তার প্রতিনিধির স্বাক্ষর তারিখ

যদি রাজি না থাকে বিস্তাড়িত লিখুন:

Annex 4: মহিলা যৌন সঙ্গীর পরীক্ষা পূর্ববর্তী কাউন্সেলিং (গোপনীয়)

একক আইডি: | | | | | |

ব্যক্তিগত কৌশল মুল্যায়ন: (দয়াকরে বৃত্তাকার করুন)	
ক্লায়েন্ট তার বিষন্নতার ইতিহাস নিদের্শ করে	পূর্বের ইতিহাস ১ বর্তমান ইতিহাস ২ কিছুই না ৩
ক্লায়েন্টের উদ্বেগের ইতিহাস নিদের্শ করে	পূর্বের ইতিহাস ১ বর্তমান ইতিহাস ২ কিছুই না ৩
ক্লায়েন্টের অন্যান্য মানসিক ব্যাধির ইতিহাস নিদের্শ করে	পূর্বের ইতিহাস ১ বর্তমান ইতিহাস ২ কিছুই না ৩
এইচআইভি পরীক্ষার ফলাফল যদি পজিটিভ হয় তাহলে সে আত্মহত্যার অভিপ্রায় ব্যক্ত করেছে কিনা	হ্যাঁ ১ না ২
ক্লায়েন্টের পুর্বে আত্মহত্যা চেষ্টার কোন ইতিহাস আছে কিনা	হ্যাঁ ১ না ২
ক্লায়েন্টের পুর্বে নিজের কোন ক্ষতি করার ইতিহাস আছে কিনা	হ্যাঁ ১ না ২
এইচআইভি পরীক্ষার ফলাফল যদি পজিটিভ হয় তাহলে ক্লায়েন্ট অন্যের কোন ক্ষতি করার অভিপ্রায় ব্যক্ত করেছে কিনা	হ্যাঁ ১ না ২<br>নির্দিষ্ট করুন:
ক্লায়েন্ট সঙ্গীকে কোন ধরনের তথ্য প্রকাশের পর সম্ভাব্য অনাকাংখিত ঘটনা কি হতে পারে তা ব্যক্ত করেছে কিনা	হ্যাঁ ১ না ২<br>নির্দিষ্ট করুন:
ক্লায়েন্টের পর্যাপ্ত ব্যক্তিগত নেটওয়ার্ক আছে	হ্যাঁ ১ না ২<br>নির্দিষ্ট করুন:

পরীক্ষা পূর্ববর্তী কাউন্সেলিং-এর সময় কাউন্সিলর কাছ থেকে সাটিফিকেট (যথাযথ বাক্সে টিক দিন)

আমি এই প্রত্যায়ন করিতেছি যে, কাউন্সিলর এর সময় নিম্নলিখিত বিষয়ে সম্পাদিত করা হবে:

- ☐ ক্লায়েন্টের সাথে আপনার ভূমিকা এবং সম্পর্ক পরিচয় করিয়ে দেন, কাজের সেবা রেকর্ড পালন সম্পর্কে ব্যাখ্যা প্রদান
- ☐ ক্লায়েন্টকে তার গোপনীয়তা রক্ষার কথা অফার করতে পারেন
- ☐ সেরো অবস্থা জানতে এবং শিখতে ক্লায়েন্টেকে প্রস্তুত করা
- ☐ এইচআইভি পরীক্ষার ফলাফল পজিটিভ হলে ক্লায়েন্ট কি করতে পারে এবং তা মোকাবেলা করার জন্য সম্ভাব্য উপায় গুলি তৈরি করা। এমন কিছু নির্দেশিত হলে এটি আত্মহত্যার ঝুঁকি মূল্যায়নে অন্তর্ভুত হতে পারে।
- ☐ পরিবার এবং বন্ধুদের কাছ থেকে সম্ভাব্য সমর্থন তৈরি করা
- ☐ পরীক্ষা এবং ফলাফল বিধান পদ্ধতি সম্পর্কে মৌলিক তথ্য তৈরি করা

<u>**অন্যান্য মন্তব্য: (প্রয়োজন হলে)**</u>

______________________________ ______________________________ ______________________________

কাউন্সিলরের নাম **কাউন্সিলরের স্বাক্ষর** **তারিখ:**

Annex 5: Quantitative questionnaire to assess HIV risk behaviours and vulnerabilities among female sex partner of HIV positive MWID in Dhaka, 2019 (Bengali)

একক আইডি: | | | | | |

এইচআইভি পজিটিভ পিডব্লিউআইডি এর মহিলা যৌনসঙ্গীর আচরণগত ঝুঁকি পরিমাপের প্রশ্নমালা, ২০১৯

জনসংখ্যা গোষ্ঠী	কোড (বৃত্ত আঁকুন যেখানে প্রযোজ্য)
স্ত্রী	১
মহিলা যৌন কর্মী	২
বিবাহিত পিডব্লিউআইডি এর অন্যান্য যৌন সঙ্গী (স্ত্রী এবং মহিলা যৌন কর্মী ব্যতীত)	৩
অবিবাহিত পিডব্লিউআইডি এর অন্যান্য যৌন সঙ্গী (মহিলা যৌন কর্মী ব্যতীত)	৪

থানার নাম: ------------------------------------- থানা কোড: | | |

সাক্ষাৎকার গ্রহণকারীর কোড: | | সাক্ষাৎকার গ্রহণকারীর নাম: ....

সাক্ষাৎকার গ্রহণকরার তারিখ : .... (দিন/মাস/বছর)

সাক্ষাৎকার শুরু করার সময় : .... (ঘন্টা ঃ মিনিট) (২৪ ঘন্টায়)

সাক্ষাৎকার শেষ করার সময় : .... (ঘন্টা ঃ মিনিট) (২৪ ঘন্টায়)

সাক্ষাৎকার গ্রহণ কি সম্পন্ন হয়েছে?

হ্যাঁ | |

না | |

যদি না হয়, কেন ? (একটি মাত্র উত্তর গ্রহণযোগ্য) :..

সুপারভাইজারের স্বাক্ষর .... তারিখ ঃ ....../....../........

সুপারভাইজারের কোড ঃ | | (দিন/মাস/বছর)

সেকশন ১ ঃ পটভূমি
(এই বিভাগের প্রশ্নাবলী সবাইকে জিজ্ঞেস করুন)

প্রশ্ন নং	প্রশ্ন	কোডের ধরন	নির্দেশ	মন্তব্য
১০১	আপনার বয়স কত? (পূর্ণ বছরে লিখুন)	বৎসর		
১০২	আপনি কত শ্রেনী পর্যন্ত লেখাপড়া শেষ করেছেন?	কখনও স্কুলে যাই নাই ৯৯<br>সম্পন্নকৃত বৎসর ......<br>১ বৎসরের কম ০০<br>শুধুমাত্র স্বাক্ষর করতে পারে ৯৬<br>জানি না/মনে নেই ৯৭<br>উত্তর না দেয়া ৯৮		
১০৩	বর্তমানে আপনি কোন ধরনের এলাকায় বাস করেন?<br>(বেশীরভাগ সময়)	আবাসিক এলাকায় ১<br>বস্তিতে ২<br>রাস্তায় ৩<br>অন্যান্য............................ ৪		
১০৪	বর্তমানে কার সাথে বসবাস করেন ?<br>(বেশীরভাগ সময়)	একা থাকি ১<br>আত্মীয়দের সাথে ২<br>বন্ধুর সাথে ৩<br>কোন ঠিকানা ছাড়া (রাস্তায়) ৪<br>PWID বন্ধুর সাথে ৫<br>PWID স্বামীর সাথে ৬<br>অন্যান্য............................ ৭		
১০৫	গত মাসে আপনার মোট আয় কত?	টাকা ....<br>জানি না/মনে নেই ৯৭<br>উত্তর না দেয়া ৯৮		
১০৬	অর্থ উপার্জনের জন্য আপনি কি করেন?<br><br>(পড়ে শোনাবেন না)<br>(একাধিক উত্তর সম্ভব)<br>(র‍্যাংকিং করুন)	র‍্যাংকিং<br>গৃহিনী ১....<br>দোকান/ক্ষুদ্র ব্যবসা ২...<br>গামেন্টসে কাজ ৩....<br>বাসায় কাজ ৪....<br>দোকানে কাজ ৫....<br>যৌন কাজ ৬....<br>অন্যান্য .... ১০...<br>জানি না/মনে নেই ৯৭<br>উত্তর না দেয়া ৯৮		

সেকশন ২ ঃ বিবাহ, সঙ্গী এবং যৌন ইতিহাস সংক্রান্ত প্রশ্নাবলী
(এই বিভাগের প্রশ্নাবলী সবাইকে জিজ্ঞেস করুন)

প্রশ্ন নং	প্রশ্ন	কোডের ধরন	নির্দেশ	মন্তব্য
২০১	বর্তমানে আপনার বৈবাহিক অবস্থা কি?<br><br>(একটি মাত্র উত্তর হবে)	বিবাহিতা ১<br>অবিবাহিতা ২<br>ডিভোর্সড ৩<br>বিধবা ৪<br>আলাদা বসবাস ৫<br>বিবাহ বর্হিভূত একসাথে বসবাস ৬<br>উত্তর না দেয়া ৯৮	(২ →) ২০৭	

প্রশ্ন নং	প্রশ্ন	কোডের ধরন	নির্দেশ	মন্তব্য
২০২	আপনি যখন প্রথম বিয়ে করেছিলেন তখন আপনার বয়স কত ছিল?	বৎসর ……. জানি না/মনে নেই ৯৭ উত্তর না দেয়া ৯৮		
২০৩	বর্তমানে আপনি কি স্বামীর সাথে বসবাস করেন?	হ্যাঁ ১ না ২		
২০৪	বর্তমানে আপনার কতজন জীবিত সন্তান আছে? (হিজড়াদের জন্য প্রযোজ্য নয়)	শূণ্য ০ কতজন ……. উত্তর না দেয়া ৯৮	→ ২০৬	
২০৫	আপনার সন্তানের বয়স কত? (সম্পুর্ণ বৎসর) (হিজড়াদের জন্য প্রযোজ্য নয়)	১ম সন্তান: বৎসর……. মাস……. ২য় সন্তান: বৎসর……. মাস……. ৩য় সন্তান: বৎসর……. মাস……. ৪র্থ সন্তান: বৎসর……. মাস…….		
২০৬	আপনি কি বর্তমানে প্রেগনেন্ট (অন্তসত্ত্বা)? (হিজড়াদের জন্য প্রযোজ্য নয়)	হ্যাঁ ১ না ২ কত মাস যাবত ----		
২০৭	কত বৎসর বয়সে আপনি প্রথম যৌনমিলন (যোনীপথে) করেছেন? (হিজড়াদের জন্য প্রযোজ্য নয়)	বৎসর ……. জানি না/মনে নেই ৯৭ উত্তর না দেয়া ৯৮		
২০৮	শেষবার যোনিপথে যৌনমিলনের সময় কনডম ব্যবহার করেছিলেন কি? (হিজড়াদের জন্য প্রযোজ্য নয়)	হ্যাঁ ১ না ২ কখনও কনডম ব্যবহার করিনি ৩ জানি না/মনে নেই ৯৭ উত্তর না দেয়া ৯৮		
২০৯	আপনি কি গত পাঁচ বছরের মধ্যে যেকোন কারণে রক্তগ্রহণ করেছেন?	হ্যাঁ ১ না ২ } জানি না/মনে নেই ৯৭ } উত্তর না দেয়া ৯৮ }	→ ২১১	
২১০	যদি হ্যাঁ হয়, তাহলে কী কারণে?			
২১১	উল্কি আঁকার ইতিহাস আছে কিনা?	হ্যাঁ ১ না ২		

সেকশন ৩ ঃ মাদক এবং ইনজেকশনের মাধ্যমে মাদকগ্রহণ সংক্রান্ত তথ্য

প্রশ্ন নং	প্রশ্ন	কোডের ধরন	নির্দেশ	মন্তব্য
৩০১	গত ১২ মাসে আপনি নেশার জন্য কি কোন মাদকদ্রব্য নিয়েছেন?	হ্যাঁ ১ না ২ } জানি না/মনে নেই ৯৭ } উত্তর না দেয়া ৯৮ }	৩০৬	
৩০২	যদি হ্যাঁ হয়, তবে আপনি কি কি মাদক দ্রব্য নিয়েছেন? <br>(পড়ে শোনাবেন না) (একাধিক উত্তর সম্ভব) (উলেখ করলে ১ -এ গোল করুন) (উলেখ না করলে ২-এ গোল করুন)	ঘুমের ট্যাবলেট ১ ২ গাঁজা ১ ২ মদ ১ ২ ফেন্সিডিল ১ ২ হেরোইন ১ ২ ইঞ্জেকশন (ব্রুপেনরফিন/পেথেডিন) ১ ২ ইয়াবা ১ ২ <br>অন্যান্য ---------------------------- ১ ২		

প্রশ্ন নং	প্রশ্ন	কোডের ধরন	নির্দেশ	মন্তব্য
৩০৩ক	আপনি সর্বশেষ কবে যে কোন মাদক নিয়েছেন?	এক মাসের মধ্যে ০<br>কত মাস আগে ....<br>জানি না/মনে নেই ৯৭<br>উত্তর না দেয়া ৯৮		
৩০৩খ	কত দিন/বছর যাবৎ আপনি যে কোন ধরনের মাদকদ্রব্য ব্যবহার করছেন?	বৎসর ....<br>মাস ....<br>এক মাসের কম ০<br>জানি না/মনে নেই ৯৭<br>উত্তর না দেয়া ৯৮		
৩০৪	আপনি কেন মাদক গ্রহন করেন?<br>(সর্বোচ্চ ৩টি)	১. ----------------------<br>২. ----------------------<br>৩. ---------------------		
৩০৫	আপনাকে কে মাদক নিতে অনুপ্রানিত করেছে?<br>(সর্বোচ্চ ৩টি)	১. ---------------------<br>২. ---------------------<br>৩. --------------------		
৩০৬	গত ১২ মাসে আপনি কি নেশা করার জন্য কোন ইনজেকশন নিয়েছেন?	হ্যাঁ ১<br>না ২<br>জানি না /মনে নাই ৯৭<br>উত্তর না দেয়া ৯৮	 → ৩১৯<br>→ ৩১৯<br>→ ৩১৯	
৩০৭	কত দিন যাবত আপনি ইনজেকশন নিয়ে নেশা করেন?	বৎসর-------মাস-------<br>জানি না/মনে নেই ৯৭<br>উত্তর না দেয়া ৯৮		
৩০৮	শেষবার কখন আপনি ইনজেকশন নিয়েছেন?	এক মাসের মধ্যে ০<br>কত মাস আগে ....<br>জানি না/মনে নেই ৯৭<br>উত্তর না দেয়া ৯৮		
৩০৯	গত ৭ দিনে আপনি কতবার ইনজেকশন নিয়েছেন?	শূন্য ০<br>সংখ্যা .....<br>জানি না/মনে নেই ৯৭<br>উত্তর না দেয়া ৯৮		
৩১০	গত সাত দিনে আপনি যতবার ইনজেকশন নিয়েছেন তার মধ্যে কতবার শেয়ার করেছেন?	শূন্য ০<br>কতবার নিয়েছেন ....<br>কতবার দিয়েছেন ....<br>জানি না/মনে নেই ৯৭<br>উত্তর না দেয়া ৯৮		
৩১১	আপনি কেন ইনজেকশন গ্রহন করেন?	১. ----------------------<br>২. ----------------------<br>৩. ---------------------		

প্রশ্ন নং	প্রশ্ন	কোডের ধরন	নির্দেশ	মন্তব্য
৩১২	আপনাকে কে ইনজেকশন নিতে অনুপ্রানিত করেছে?	১. ----------------------<br>২. ----------------------<br>৩. ----------------------		
৩১৩	শেষবার (গত ১২ মাসের মধ্যে) নেশার ইনজেকশন নেয়ার সময় আপনি কি আপনার ব্যবহৃত সুচঁ সিরিঞ্জ আপনার **স্বামী/বন্ধু/যৌন সঙ্গী** ব্যবহার করার পরে ব্যবহার করেছেন?	হ্যাঁ ১<br>না ২<br>জানি না /মনে নাই ৯৭<br>উত্তর না দেয়া ৯৮		
৩১৪	শেষবার (গত ১২ মাসের মধ্যে) আপনার ব্যবহৃত সুচঁ সিরিঞ্জ আপনার **স্বামী/বন্ধু/যৌন সঙ্গীর** কাছে পাস করেছেন?	হ্যাঁ ১<br>না ২<br>জানি না /মনে নাই ৯৭<br>উত্তর না দেয়া ৯৮		
৩১৫	শেষবার (গত ১২ মাসের মধ্যে) আপনি কি সুচঁ সিরিঞ্জ আপনার **স্বামী/বন্ধু/যৌন সঙ্গী** ছাড়া অন্য কেউ ব্যবহার করার পর আপনি ব্যবহার করেছেন?	হ্যাঁ ১<br>না ২<br>জানি না /মনে নাই ৯৭<br>উত্তর না দেয়া ৯৮		
৩১৬	শেষবার (গত ১২ মাসের মধ্যে) নেশার ইনজেকশন নেয়ার সময় আপনি কি আপনার ব্যবহৃত সুচঁ সিরিঞ্জ আপনার **স্বামী/বন্ধু/যৌন সঙ্গী ছাড়া** অন্য কারো কাছে পাস করেছেন?	হ্যাঁ ১<br>না ২<br>জানি না /মনে নাই ৯৭<br>উত্তর না দেয়া ৯৮		
৩১৭	আপনি কি জানেন কোন জায়গায় যেখানে নতুন সুচঁ সিরিঞ্জ পাওয়া যায়?	হ্যাঁ ১<br>না ২<br>জানি না /মনে নাই ৯৭<br>উত্তর না দেয়া ৯৮	(না/জানি না/উত্তর না দেয়া) → ৩১৯	
৩১৮	আপনি কোথা থেকে নতুন সূঁচ এবং সিরিঞ্জ পান?<br><br>(পড়ে শোনাবেন না)<br>(একাধিক উত্তর হতে পারে।)<br>(উল্লেখ করলে ১ গোল করুন)<br>(উল্লেখ না করলে ২ গোল করুন)	ফার্মেসী ১ ২<br>বন্ধুবান্ধব ১ ২<br>ড্রাগ গ্রহণকারী সঙ্গী ১ ২<br>এনজিও কর্মী ১ ২<br>ড্রাগ বিক্রেতা ১ ২<br>অন্যান্য .... ১ ২<br>জানি না/মনে নেই ৯৭<br>উত্তর না দেয়া ৯৮		
৩১৯	আপনি কি OST কর্মসূচীর সাথে যুক্ত আছেন? (মধু/মেথাডন/ঔষধ)	হ্যাঁ ১<br>না ২<br>জানি না/মনে নেই ৯৭<br>উত্তর না দেয়া ৯৮	 → ৪০১<br>→ ৪০১<br>→ ৪০১	
৩২০	যদি হ্যাঁ হয়, কতদিন ধরে যুক্ত আছেন?	মাস ....<br>১ মাসের মধ্যে ০<br>জানি না/মনে নাই ৯৭<br>উত্তর না দেয়া ৯৮		

সেকশন ৪ঃ স্বামীর সাথে যৌনআচরণ

(এখন আমি আপনাকে স্বামীর সাথে যৌনকাজের সম্পর্কে জিজ্ঞাসা করবো, যতটুকু আপনি মনে করতে পারেন)

প্রশ্ন নং	প্রশ্ন	কোডের ধরন	নির্দেশ	মন্তব্য
৪০১	আপনি সর্বশেষ কবে আপনার স্বামীর সাথে যৌনমিলন করেছিলেন?	১ মাসের মধ্যে ০<br>------------------- মাস আগে<br>অবিবাহিত ৯৬<br>জানি না/মনে নাই ৯৭<br>উত্তর না দেওয়া ৯৮	১২ মাস আগে হলে ৪০৮ এ চলে যান।	

প্রশ্ন নং	প্রশ্ন	কোডের ধরন	নির্দেশ	মন্তব্য
৪০২ক	আপনি গত **১২** মাসের মধ্যে আপনার স্বামীর সাথে কতবার যোনীপথে যৌনমিলন করেছেন?	শূন্য ০<br>বার .....<br>জানি না/মনে নাই ৯৭<br>উত্তর না দেওয়া ৯৮	→ ৪০৪	
৪০২খ	গত **১২** মাসে আপনার স্বামীর সাথে যোনীপথে যৌনমিলনের সময় কি হারে কনডম ব্যবহার করেছেন। (১-৩ পড়ে শুনান)	সব সময় ১<br>মাঝে মাঝে ২<br>কখনো না ৩<br>উত্তর না দেয়া ৯৮		
৪০৩	গত **১২** মাসে শেষবার আপনার স্বামীর সাথে যোনীপথে যৌনমিলনের সময় কি কনডম ব্যবহার করেছিলেন?	হ্যাঁ ১<br>না ২<br>জানি না/মনে নাই ৯৭<br>উত্তর না দেয়া ৯৮		
৪০৪	গত **১২** মাসে আপনার স্বামীর সাথে মুখে যৌনমিলন করেছিলেন?	বীর্যপাত পর্যন্ত ১<br>বীর্যপাতের আগ পর্যন্ত ২<br>না ৩<br>জানি না/মনে নাই ৯৭<br>উত্তর না দেয়া ৯৮	(২-৯৮) → ৪০৬	
৪০৫	যদি হ্যাঁ, আপনি গত **১২** মাসে কি হারে কনডম ব্যবহার করেছিলেন?	সব সময় ১<br>মাঝে মাঝে ২<br>কখনো না ৩<br>উত্তর না দেয়া ৯৮		
৪০৬	গত **১২** মাসে আপনার স্বামীর সাথে পায়ু পথে সেক্স করেছিলেন?	হ্যাঁ ১<br>না ২<br>জানি না/মনে নাই ৯৭<br>উত্তর না দেয়া ৯৮	(২-৯৮) → ৪০৮	
৪০৭	যদি হ্যাঁ হয়, তাহলে আপনি গত **১২** মাসে কি হারে কনডম ব্যবহার করেছেন?	সব সময় ১<br>মাঝে মাঝে ২<br>কখনো না ৩<br>উত্তর না দেয়া ৯৮		
৪০৮	আপনার স্বামী মজা করার জন্য বা নেশা করার জন্য ইঞ্জেকশনের মাধ্যমে মাদকদ্রব্য নেয়?	হ্যাঁ ১<br>না ২<br>জানি না/মনে নাই ৯৭<br>উত্তর না দেয়া ৯৮		

সেকশন ৫ঃ মহিলা যৌনকর্মীর যৌনআচরণ

(এখন আমি আপনাকে পুরুষ যৌনসঙ্গীর সাথে যৌনকাজের সম্পর্কে জিজ্ঞাসা করবো, যতটুকু আপনি মনে করতে পারেন)

প্রশ্ন নং	প্রশ্ন	কোডের ধরন	নির্দেশ	মন্তব্য
৫০১	আপনি সর্বশেষ কবে খদ্দেরের সাথে যৌনমিলন করেছিলেন?	১ মাসের মধ্যে ০<br>------------------- মাস আগে<br>১ সপ্তাহের মধ্যে ৯৬<br>জানি না/মনে নাই ৯৭<br>উত্তর না দেওয়া ৯৮<br>কখনো না ৯৯	১২ মাস আগে হলে ৬০১ এ চলে যান।	
৫০২	গত **১২** মাসে আপনি কতজন ভিন্ন ভিন্ন পুরুষের সাথে টাকা নিয়ে **(যোনীপথে)** যৌনমিলন করেছেন?	সংখ্যা ......<br>জানি না/মনে নেই ৯৭<br>উত্তর না দেয়া ৯৮		
৫০৩	গত **১২** মাসে আপনি **কতবার** টাকা নিয়ে পুরুষের সাথে **(যোনীপথে)** যৌনমিলন করেছেন?	শূন্য ০<br>সংখ্যা ....<br>জানি না/মনে নেই ৯৭	→ ৫০৬	

প্রশ্ন নং	প্রশ্ন	কোডের ধরন	নির্দেশ	মন্তব্য
		উত্তর না দেয়া ৯৮		
৫০৪	গত ১২ মাসে আপনার সকল খদ্দেরের সাথে যোনীপথে যৌনমিলনের সময় কি হারে কনডম ব্যবহার করেছিলেন?<br>(১-৩ পড়ে শুনান)	সব সময় ১<br>মাঝে মাঝে ২<br>কখনো না ৩<br>গত ১২ মাসে যৌনকাজ করি নাই ৯৬<br>উত্তর না দেয়া ৯৮		
৫০৫	গত ১২ মাসে শেষবার পুরুষ খদ্দেরের সাথে যোনীপথে যৌনমিলনের সময় কি কনডম ব্যবহার করেছিলেন?	হ্যাঁ ১<br>না ২<br>গত ১২ মাসে যৌনকাজ করি নাই ৯৬<br>জানি না/মনে নাই ৯৭<br>উত্তর না দেয়া ৯৮		
৫০৬	গত ১২ মাসে আপনার খদ্দেরের সাথে মুখে যৌনমিলন করেছিলেন?	বীর্যপাত পর্যন্ত ১<br>বীর্যপাতের আগ পর্যন্ত ২<br>না ৩<br>গত ১২ মাসে যৌনকাজ করি নাই ৯৬<br>জানি না/মনে নাই ৯৭<br>উত্তর না দেয়া ৯৮	(২–৯৮) → ৫০৮	
৫০৭	যদি হ্যাঁ, আপনি গত ১২ মাসে কি হারে কনডম ব্যবহার করেছিলেন?	সব সময় ১<br>মাঝে মাঝে ২<br>কখনো না ৩<br>উত্তর না দেয়া ৯৮		
৫০৮	গত ১২ মাসে আপনার খদ্দেরের সাথে পায়ু পথে সেক্স করেছেন?	হ্যাঁ ১<br>না ২<br>গত ১২ মাসে যৌনকাজ করি নাই ৯৬<br>জানি না/মনে নাই ৯৭<br>উত্তর না দেয়া ৯৮	(২–৯৮) → ৫১০	
৫০৯	যদি হ্যাঁ হয়, তাহলে আপনি গত ১২ মাসে কি হারে কনডম ব্যবহার করেছেন?	সব সময় ১<br>মাঝে মাঝে ২<br>কখনো না ৩<br>উত্তর না দেয়া ৯৮		
৫১০	আপনার কোন খদ্দের (নতুন বা নিয়মিত) মজা করার জন্য বা নেশা করার জন্য ইঞ্জেকশনের মাধ্যমে মাদকদ্রব্য নেয়?	হ্যাঁ ১<br>না ২<br>জানি না/মনে নাই ৯৭<br>উত্তর না দেয়া ৯৮		

সেকশন ৬ঃ টাকা ছাড়া (স্বামী ব্যতিত) যৌন সঙ্গীর সাথে যৌনআচরণ

(এখন আমি আপনাকে টাকা ছাড়া পুরুষ যৌনসঙ্গীর সাথে যৌনকাজের সম্পর্কে জিজ্ঞাসা করবো, যতটুকু আপনি মনে করতে পারেন)

প্রশ্ন নং	প্রশ্ন	কোডের ধরন	নির্দেশ	মন্তব্য
৬০১	আপনি সর্বশেষ কবে টাকা ছাড়া পুরুষ যৌন সঙ্গীর সাথে যৌনমিলন করেছিলেন?	১ মাসের মধ্যে ০<br>------------------- মাস আগে<br>না ২<br>জানি না/মনে নাই ৯৭<br>উত্তর না দেওয়া ৯৮<br>কখনো না ৯৯	(২–৯৯) → ৭০১	
৬০২	গত ১২মাসে আপনি কতবার টাকা ছাড়া যৌনসঙ্গীর সাথে (যোনীপথে) যৌনমিলন করেছেন?	শূন্য ০<br>সংখ্যা ......<br>জানি না/মনে নেই ৯৭	(শূন্য) → ৬০৫	

প্রশ্ন নং	প্রশ্ন	কোডের ধরন	নির্দেশ	মন্তব্য
		উত্তর না দেয়া ৯৮		
৬০৩	গত ১২ মাসে আপনার টাকা ছাড়া সকল পুরুষ যৌনসঙ্গীর সাথে যোনীপথে যৌনমিলনের সময় কি হারে কনডম ব্যবহার করেছিলেন?<br>(১-৩ পড়ে শুনান)	সব সময় ১<br>মাঝে মাঝে ২<br>কখনো না ৩<br>উত্তর না দেয়া ৯৮		
৬০৪	গত ১২ মাসে শেষবার টাকা ছাড়া পুরুষ যৌনসঙ্গীর সাথে যোনীপথে যৌনমিলনের সময় কি কনডম ব্যবহার করেছিলেন?	হ্যাঁ ১<br>না ২<br>জানি না/মনে নাই ৯৭<br>উত্তর না দেয়া ৯৮		
৬০৫	গত ১২ মাসে আপনার টাকা ছাড়া পুরুষ যৌন সঙ্গীদের সাথে মুখে (বীর্যপাত পর্যন্ত) যৌনমিলন করেছিলেন?	বীর্যপাত পর্যন্ত ১<br>বীর্যপাতের আগ পর্যন্ত ২<br>না ৩<br>জানি না/মনে নাই ৯৭<br>উত্তর না দেয়া ৯৮	(২, ৩, ৯৭, ৯৮) → ৬০৭	
৬০৬	যদি হ্যাঁ, আপনি গত ১২ মাসে কি হারে কনডম ব্যবহার করেছিলেন?	সব সময় ১<br>মাঝে মাঝে ২<br>কখনো না ৩<br>উত্তর না দেয়া ৯৮		
৬০৭	গত ১২ মাসে আপনার টাকা ছাড়া পুরুষ যৌন সঙ্গী সঙ্গে পায়ু পথে সেক্স করেছেন?	হ্যাঁ ১<br>না ২<br>জানি না/মনে নাই ৯৭<br>উত্তর না দেয়া ৯৮	(২, ৯৭, ৯৮) → ৬০৯	
৬০৮	যদি হ্যাঁ হয়, তাহলে আপনি গত ১২ মাসে কি হারে কনডম ব্যবহার করেছেন?	সব সময় ১<br>মাঝে মাঝে ২<br>কখনো না ৩<br>উত্তর না দেয়া ৯৮		
৬০৯	আপনার টাকা ছাড়া (স্বামী ব্যতিত) কোন যৌন সঙ্গী মজা করার জন্য বা নেশা করার জন্য ইঞ্জেকশনের মাধ্যমে মাদকদ্রব্য নেয়?	হ্যাঁ ১<br>না ২<br>জানি না/মনে নাই ৯৭<br>উত্তর না দেয়া ৯৮		

সেকশন ৭ঃ যৌনরোগ সম্পর্কিত

(এই বিভাগের প্রশ্নাবলী সবাইকে জিজ্ঞেস করুন। এখন আমি আপনাকে যৌনরোগ সম্পর্কে জিজ্ঞাসা করবো)

প্রশ্ন নং	প্রশ্ন	কোডের ধরন	নির্দেশ	মন্তব্য
৭০১	আপনি কি আমাকে মহিলাদের এমন কোন রোগের লক্ষণ বা উপসর্গের কথা বলতে পারেন যা যৌনমিলনের (যোনীপথে/পায়ুপথে/মুখে) মাধ্যমে ছড়ায়?<br><br>(পড়ে শোনাবেন না)<br>(উল্লেখ করলে ১-এ গোল করুন)<br>(উল্লেক না করলে ২-এ গোল করুন)<br>(একাধিক উত্তর সম্ভব)	যোনীপথে স্রাব ১ ২<br>গন্ধযুক্ত স্রাব ১ ২<br>যৌনাঙ্গে ঘাঁ/ক্ষত ১ ২<br>তলপেটে ব্যথা ১ ২<br>অন্যান্য-------------------------- ১ ২<br>জানি না/মনে নাই ৯৭<br>উত্তর না দেওয়া ৯৮		
৭০২	গত ১ বৎসরে আপনার যোনীপথে ব্যথাসহ বা দুর্গন্ধযুক্ত স্রাব ছিল কি?	হ্যাঁ ১<br>না ২<br>জানি না/মনে নাই ৯৭<br>উত্তর না দেয়া ৯৮		
৭০৩	গত ১ বৎসরে আপনার মাসিকের ব্যথা বা পেটের অসুখ ছাড়া তলপেটে ব্যথা ছিল কি?	হ্যাঁ ১<br>না ২		

প্রশ্ন নং	প্রশ্ন	কোডের ধরন	নির্দেশ	মন্তব্য
		জানি না/মনে নাই ৯৭<br>উত্তর না দেয়া ৯৮		
৭০৪	গত ১ বৎসরে আপনার যোনীপথের ভিতরে বা বাহিরে ঘাঁ/ক্ষত হয়েছিল কি?	হ্যাঁ ১<br>না ২<br>জানি না/মনে নাই ৯৭<br>উত্তর না দেয়া ৯৮		
৭০৫	প্রশ্ন নং ৭০২, ৭০৩ এবং ৭০৪ দেখুন যদি যেকোন একটিতে হ্যাঁ হয় তবে প্রশ্ন নং ৭০৫-এ ১ গোল করুন অন্যথায় ২ গোল করুন।	যে কোন একটি হ্যাঁ ১<br>সব না ২	→ ৮০১	
৭০৬	আপনি কী কোন চিকিৎসা গ্রহণ করেছেন?	হ্যাঁ ১<br>না ২<br>জানি না/মনে নাই ৯৭<br>উত্তর না দেয়া ৯৮	→ ৮০১	
৭০৭	যদি হ্যাঁ হয়, তাহলে কোথায়?<br><br>পড়ে শোনাবেন না<br>(একটি মাত্র উত্তর হবে)	সরকারী হাসপাতালে চিকিৎসা ১<br>ফামের্সী থেকে চিকিৎসা ২<br>প্রাইভেট ডাক্তার দ্বারা চিকিৎসা ৩<br>প্রাইভেট ক্লিনিক থেকে চিকিৎসা ৪<br>NGO ক্লিনিক থেকে চিকিৎসা ৫<br>এনজিও ক্লিনিকের নাম ....<br>সনাতনী চিকিৎসা ৬<br>বন্ধুবান্ধব থেকে উপদেশ ৭<br>নিজে নিজে চিকিৎসা ৮<br>অন্যান্য .... ৯<br>জানি না/মনে নাই ৯৭<br>উত্তর না দেয়া ৯৮		

সেকশন ৮ঃ এইচআইভি সমন্ধে জ্ঞান
(এখন আমি আপনাকে এইচআইভি সমন্ধে জিজ্ঞাসা করবো)

প্রশ্ন নং	প্রশ্ন	কোডের ধরন	নির্দেশ	মন্তব্য
৮০১	আপনি কি আগে কখনো HIV অথবা AIDS নামক রোগের নাম শুনেছেন?	হ্যাঁ ১<br>না ২<br>এই ডিআইসির আওতায় টেষ্ট হয়েছে ৩<br>উত্তর না দেয়া ৯৮	(না, ৩, ৯৮) → ৯০১	
৮০২	প্রতিবার যৌনমিলনের সময় সঠিকভাবে কনডম ব্যবহারের মাধ্যমে মানুষ কি HIV/AIDS -এর ঝূঁকি কমাতে পারে?	হ্যাঁ ১<br>না ২<br>জানি না/মনে নাই ৯৭<br>উত্তর না দেয়া ৯৮		
৮০৩	মশার কামড় থেকে কি কোন মানুষ HIV/AIDS দ্বারা আক্রান্ত হতে পারে?	হ্যাঁ ১<br>না ২<br>জানি না/মনে নাই ৯৭<br>উত্তর না দেয়া ৯৮		
৮০৪	HIV/AIDS দ্বারা আক্রান্ত লোকের সাথে একত্রে খাবার খেয়ে কেউ কি HIV/AIDS দ্বারা আক্রান্ত হতে পারে?	হ্যাঁ ১<br>না ২<br>জানি না/মনে নাই ৯৭<br>উত্তর না দেয়া ৯৮		
৮০৫	অন্যের ব্যবহৃত সূঁচ/সিরিঞ্জ ব্যবহার করলে কেউ কি HIV/AIDS দ্বারা আক্রান্ত হতে পারে?	হ্যাঁ ১<br>না ২<br>জানি না/মনে নাই ৯৭<br>উত্তর না দেয়া ৯৮		

প্রশ্ন নং	প্রশ্ন	কোডের ধরন	নির্দেশ	মন্তব্য
৮০৬	একাধিক যৌনসঙ্গী পরিহার করে কি কেউ HIV/AIDS -এর ঝুঁকি কমাতে পারে?	হ্যাঁ ১<br>না ২<br>জানি না/মনে নাই ৯৭<br>উত্তর না দেয়া ৯৮		
৮০৭	আপনি কি মনে করেন যে কাউকে দেখেই বলা যাবে সে HIV /AIDS দ্বারা আক্রান্ত?	হ্যাঁ ১<br>না ২<br>জানি না/মনে নাই ৯৭<br>উত্তর না দেয়া ৯৮		

সেকশন ৯ঃ গোপনীয়ভাবে এইচআইভি পরীক্ষা

(এখন আমি আপনাকে গোপনীয়ভাবে এইচআইভি পরীক্ষা সমন্ধে জিজ্ঞাসা করবো)

প্রশ্ন নং	প্রশ্ন	কোডের ধরন	নির্দেশ	মন্তব্য
৯০১	আপনি HIV দ্বারা আক্রান্ত কিনা তা যদি পরীক্ষা করে দেখতে চান তাহলে গোপনীয়ভাবে কোথায় করতে পারবেন তা কি জানেন?	হ্যাঁ ১<br>না ২<br>এই ডিআইসির আওতায় টেষ্ট হয়েছে ৩<br>জানি না/মনে নাই ৯৭<br>উত্তর না দেয়া ৯৮	 → ১০০১<br><br>→ ১০০১<br>→ ১০০১	
৯০২	(পরীক্ষার ফলাফল আমি জানতে চাইনা)<br>আপনি কি কখনো HIV পরীক্ষা করেছেন?	হ্যাঁ ১<br>না ২<br>জানি না/মনে নাই ৯৭<br>উত্তর না দেয়া ৯৮	→ ৯০৪<br>(না/জানি না/উত্তর না দেয়া) → ৯০৩	
৯০৩	আপনি কেন HIV পরীক্ষা করেন নাই?<br><br>(একাধিক উত্তর সম্ভব)<br>(পড়ে শোনাবেন না)<br>(উল্লেখ করলে ১-এ গোল করুন)<br>(উল্লেখ না করলে ২-এ গোল করুন)<br><br>(এই প্রশ্ন জিজ্ঞাসা করার পরে ১০০১ নং প্রশ্নে যান)	কেউ আমাকে বলে নাই ১ ২<br>পরিবারের লোকজন কর্তৃক অপবাদের ভয়/আশংঙ্কা ১ ২<br>NGO কর্মী কর্তৃক অপবাদের ভয়/ আশংঙ্কা ১ ২<br>প্রতিবেশী কর্তৃক অপবাদের ভয়/ আশংঙ্কা ১ ২<br>নির্যাতন/ গ্রেফতারের ভয়/ আশংঙ্কা/ অভিজ্ঞতা ১ ২<br>অন্যান্য.................................. ১ ২	→ ১০০১	
৯০৪	হ্যাঁ হলে শেষবার কোথায় HIV পরীক্ষা করেছেন?			
৯০৫	আপনি কি স্বেচ্ছায় HIV পরীক্ষা করিয়েছেন নাকি অন্য কেউ উৎসাহিত করেছে অথবা আপনার পরীক্ষা প্রয়োজন হয়েছিল ?	স্বেচছায় ১<br>অন্য কেউ উৎসাহিত করেছিল ২<br>প্রয়োজন হয়েছিল ৩<br>উত্তর না দেয়া ৯৮		
৯০৬	আমি ফলাফল জানতে চাই না, আপনি ঐ ফলাফল পেয়েছিলেন কি?	হ্যাঁ ১<br>না ২<br>জানি না/মনে নাই ৯৭<br>উত্তর না দেয়া ৯৮	(না/জানি না/উত্তর না দেয়া) → ১০০১	
৯০৭	সর্বশেষ কতদিন আগে আপনি HIV পরীক্ষা করিয়েছেন?	গত ৬ মাসের মধ্যে ০<br>৬ মাস থেকে ১ বছরের মধ্যে ১<br>১ বছর আগে ২<br>জানি না/মনে নাই ৯৭<br>উত্তর না দেয়া ৯৮		

সেকশন ১০ঃ এইচআইভি ঝুঁকি সম্পর্কিত ধারনা

(এখন আমি আপনাকে এইচআইভি এর ঝুঁকি সমন্ধে জিজ্ঞাসা করবো)

প্রশ্ন নং	প্রশ্ন	কোডের ধরন	নির্দেশ	মন্তব্য
১০০১	আপনি কি মনে করেন আপনার HIV -এর ঝুঁকি আছে?	বেশী ১<br>মাঝারী ২<br>কম ৩<br>ঝুঁকি নেই ৪<br>এইচআইভি পজিটিভ ৯৬<br>জানি না/মনে নাই ৯৭<br>উত্তর না দেয়া ৯৮	(বেশী, মাঝারী) → ১০০২<br>(কম, ঝুঁকি নেই) → ১০০৩<br>(৯৬, ৯৭, ৯৮) → ১১০১	
১০০২	আপনি কেন মনে করেন আপনার HIV হওয়ার বেশী বা মাঝারী ঝুঁকি রয়েছে?<br><br>(পড়ে শোনাবেন না)<br>(একাধিক উত্তর সম্ভব)<br>(উল্লেখ করলে ১-এ গোল করুন)<br>(উল্লেখ না করলে ২-এ গোল করুন)	ঝুঁকি পূর্ন আচরণ ১ ২<br>পুনঃপুনঃ পায়ুপথে যৌন কাজ ১ ২<br>পুনঃপুনঃ যোনীপথে যৌন কাজ ১ ২<br>মাঝে মাঝে কনডম ব্যবহার ১ ২<br>সুঁচ/সিরিঞ্জ ভাগাভাগি ১ ২<br>অন্যান্য .... ১ ২<br>জানি না/মনে নাই ৯৭<br>উত্তর না দেয়া ৯৮	এই প্রশ্নের উত্তর আসলে ১০০৩ প্রশ্ন করার দরকার নাই।	
১০০৩	আপনি কেন মনে করেন আপনার HIV হওয়ার ঝুঁকি নাই অথবা কম?<br><br>(পড়ে শোনাবেন না)<br>(একাধিক উত্তর সম্ভব)<br>(উল্লেখ করলে ১-এ গোল করুন)<br>(উল্লেখ না করলে ২-এ গোল করুন)	সর্বদা কনডম ব্যবহার ১ ২<br>পরিস্কার সঙ্গী/ খদ্দের ১ ২<br>স্বাস্থ্যবান সঙ্গী/ খদ্দের ১ ২<br>কখনও সূঁচ/সিরিঞ্জি ভাগাভাগী করি না ১ ২<br>মাঝে মাঝে সূঁচ/সিরিঞ্জি ভাগাভাগী করি ১ ২<br>অনিয়মিত কনডম ব্যবহার ১ ২<br>সব সময় একজন বিশ্বস্ত সঙ্গীর সাথে যৌনমিলন করি ১ ২<br>সবসময় পরিস্কার পরিচ্ছন্ন থাকি ১ ২<br>কম যৌনমিলন করি ১ ২<br>যৌনমিলন করার পর ধুয়ে ফেলি ১ ২<br>অন্যান্য .... ১ ২<br>জানি না/মনে নাই ৯৭<br>উত্তর না দেয়া ৯৮		

সেকশন ১১ঃ হেপাটাইটিস সি সমন্ধে জ্ঞান

(এখন আমি আপনাকে হেপাটাইটিস সি সমন্ধে জিজ্ঞাসা করবো)

প্রশ্ন নং	প্রশ্ন	কোডের ধরন	নির্দেশ	মন্তব্য
১১০১	আপনি কি আগে কখনও হেপাটাইটিস সি ভাইরাস এর কথা শুনেছেন?	হ্যাঁ ১<br>না ২<br>উত্তর না দেয়া ৯৮	→ ১২০১	
১১০২	দয়া করে বলুন কিভাবে ছড়ায়?	১. ----------------------<br>২. ----------------------<br>৩. ----------------------		

সেকশন ১২ ঃ কর্মসূচীতে অংশগ্রহণ

(এখন আমি আপনাকে এইচআইভি প্রতিরোধের সেবা সমন্ধে জিজ্ঞাসা করবো)

প্রশ্ন নং	প্রশ্ন	কোডের ধরন	নির্দেশ	মন্তব্য
১২০১	আপনি কি জীবনে কখনো এনজিও পরিচালিত কোন এইডস প্রতিরোধ কর্মসূচীতে অংশগ্রহণ করেছিলেন?	হ্যাঁ ১<br>না ২<br>জানি না/মনে নাই ৯৭<br>উত্তর না দেয়া ৯৮	(না, জানি না/মনে নাই, উত্তর না দেয়া) → ১৩০১	
১২০২	আপনি সর্বশেষ কবে এনজিও পরিচালিত কোন এইডস প্রতিরোধ কর্মসূচী থেকে সেবা নিয়েছিলেন ।	১ মাসের মধ্যে ০<br>-- -- -- -- মাস পূর্বে<br>জানি না/মনে নাই ৯৭<br>উত্তর না দেয়া ৯৮		
১২০৩	আপনি সারা জীবনে এনজিও থেকে কি কি ধরনের সেবা নিয়েছিলেন?<br><br>(পড়ে শোনাবেন না)<br>(একাধিক উত্তর গ্রহণ যোগ্য)<br>(উল্লেখ করলে ১ গোল করুন)<br>(উল্লেখ না করলে ২ গোল করুন)	সূঁচ/সিরিঞ্জ বিনিময় কর্মসূচী ১ ২<br>শিক্ষামূলক কার্যক্রম ১ ২<br>HIV পরীক্ষা করেছি ১ ২<br>কনডম পেয়েছেন ১ ২<br>যৌনরোগের জন্য চিকিৎসা পেয়েছেন ১ ২<br>সাধারন রোগের জন্য চিকিৎসা পেয়েছেন ১ ২<br>ANC তে অংশগ্রহণ ১ ২<br>ART ১ ২<br>ঘুমাইছি/গোছল করেছি ১ ২<br>টিভি/কেরাম/লুডু খেলেছি ১ ২<br>অন্যান্য.................................... ১ ২<br>জানি না/মনে নাই ৯৭<br>উত্তর না দেয়া ৯৮		

<u>সেকশন ১৩: যক্ষ্মা সম্পর্কিত চিকিৎসার ইতিহাস</u>

প্রশ্ন নং	প্রশ্ন	কোডের ধরন	নির্দেশ	মন্তব্য
১৩০১.	আপনি কি কখনো যক্ষ্মা দ্বারা আক্রান্ত হয়েছেন?	হ্যাঁ (পরীক্ষা করেছে) ১<br>না (পরীক্ষা করেছে) ২<br>কখনো পরীক্ষা করে নাই ৩<br>জানি না ৯৭<br>উওর না দেয়া ৯৮	(না, কখনো পরীক্ষা করে নাই, জানি না, উওর না দেয়া) → ১৪০১	
১৩০২	যদি 'হ্যাঁ' হয়,তবে কত বার আক্রান্ত হয়েছেন?	বার ------		
১৩০৩	যদি 'হ্যাঁ' হয়, তবে সর্বশেষ যক্ষ্মার ধরন	ফুসফুসের যক্ষ্মা (পালমোনারী যক্ষ্মা) ১<br>ফুসফুস ব্যাতিত অন্য স্থানের যক্ষা (এক্সট্রা পালমোনারী যক্ষ্মা) ২<br>স্থান নির্দিষ্ট করুন--------		
১৩০৪	আপনি কি যক্ষ্মার জন্য কোন চিকিৎসা নিয়েছেন?	হ্যাঁ ১<br>না ২<br>জানি না ৯৭<br>উওর না দেয়া ৯৮	(না, জানি না, উওর না দেয়া) → ১৪০১	
১৩০৫	যদি 'হ্যাঁ' হয়, সর্বশেষ / সবচেয়ে সাম্প্রতিক চিকিৎসার 'রেজিমেন'?	৬ মাস ব্যাপী যক্ষ্মার মুখে খাওয়ার ড্রাগ (ক্যাটাগরী ১) ১<br>৮ মাস ব্যাপী যক্ষার মুখে খাওয়ার ড্রাগ এবং ২ মাস ইনজেকশন ড্রাগ (ক্যাটাগরী ২) ২		

প্রশ্ন নং	প্রশ্ন	কোডের ধরন	নির্দেশ	মন্তব্য
১৩০৬	যদি 'হ্যাঁ' হয়, তবে কি ফলাফল ছিল?	বর্তমানে চিকিৎসারত ১<br>নিরাময় (চিকিৎসা সম্পূর্ণ এবং ফলাফল নেগেটিভ) ২<br>চিকিৎসা সম্পূর্ণ (চিকিৎসা সম্পূর্ণ কিন্তু পরীক্ষা এখনও হয়নি) ৩<br>চিকিৎসায় ব্যর্থ (চিকিৎসা সম্পূর্ণ কিন্তু ফলাফল পজিটিভ) ৪<br>ফলোআপ থেকে হারিয়ে গেছে (চিকিৎসা শুরু করেনি/২ মাসের বেশি ঔষধ বন্ধ আছে) ৫<br>অন্যান্য (নির্দিষ্ট করুন---------) ৬		
১৩০৭	আপনি সর্বশেষ বার কোথা থেকে যক্ষ্মার চিকিৎসা পেয়েছেন? (একটি মাত্র উত্তর দিতে হবে)	সরকারী / বক্ষ্যব্যাধি হাসপাতালে মহাখালী) ১<br>সরকারী টিবি ক্লিনিক ২<br>উপজেলা স্বাস্থ্য কেন্দ্র ৩<br>BRAC এর DOTS কেন্দ্র ৪<br>নগর স্বাস্থ্য কেন্দ্র ৫<br>সূর্যের হাসি ৬<br>ব্লুস্টার (SMC) ৭<br>যক্ষা নির্ণয় ও চিকিৎসা কেন্দ্র, ৮<br>আইসিডিডিআর,বি ৯<br>অন্যান্য((নির্দিষ্ট করুন----) ৯৭		

সেকশন ১৪ ঃ নির্যাতন সংক্রান্ত তথ্য

**প্রশ্ন নং**	**প্রশ্ন**	**কোডের ধরন**	**নির্দেশ**	**মন্তব্য**
১৪০১	গত ১২ মাসে কেউ কি আপনাকে মারধোর করেছে?	হ্যাঁ ১<br>না ২<br>জানি না/মনে নাই ৯৭<br>উত্তর না দেয়া ৯৮	(না/৯৭/৯৮) → ১৪০৩	
১৪০২	গত ১২ মাসে কে আপনাকে মারধোর করেছে?<br><br>**(পড়ে শোনাবেন না)**<br>**(একাধিক উত্তর সম্ভব)**<br>**(উল্লেখ করলে ১-এ গোল করুন)**<br>**(উল্লেখ না করলে ২-এ গোল করুন)**	আইন-শৃঙ্খলা বাহিনী ১ ২<br>মাস্তান ১ ২<br>নতুন খদ্দের ১ ২<br>নিয়মিত খদ্দের ১ ২<br>মহল্লার লোক ১ ২<br>ভালবাসার মানুষ ১ ২<br>পরিবার/ আত্মীয় ১ ২<br>(সম্পর্ক)...............................		পরিবার / আত্মীয়র ক্ষেত্রে সম্পর্কের নাম উল্লেখ করতে হবে
১৪০৩	গত ১২ মাসে কেউ কি আপনার ইচ্ছার বিরুদ্ধে যৌনমিলন করেছে?	হ্যাঁ ১<br>না ২<br>জানি না/মনে নাই ৯৭<br>উত্তর না দেয়া ৯৮	(না/৯৭/৯৮) → ধন্যবাদ দিয়ে শেষ করুন	
১৪০৪	গত ১২ মাসে কে আপনার ইচ্ছার বিরুদ্ধে জোরপূর্বক যৌনমিলন করেছে?<br><br>**(পড়ে শোনাবেন না)**<br>**(একাধিক উত্তর সম্ভব)**<br>**(উল্লেখ করলে ১-এ গোল করুন)**<br>**(উল্লেখ না করলে ২-এ গোল করুন)**	আইন-শৃঙ্খলা বাহিনী ১ ২<br>মাস্তান ১ ২<br>নতুন খদ্দের ১ ২<br>নিয়মিত খদ্দের ১ ২<br>মহল্লার লোক ১ ২<br>ভালবাসার মানুষ ১ ২<br>পরিবার/ আত্মীয় ১ ২<br>(সম্পর্ক)		পরিবার / আত্মীয়র ক্ষেত্রে সম্পর্কের নাম উল্লেখ করতে হবে

*** আপনার মূল্যবান সময় দিয়ে সহযোগীতা করার জন্য অসংখ্য ধন্যবাদ।**

Annex 6: এইচআইভি পজিটিভ পিডব্লিউআইডি মহিলা যৌন সঙ্গীদের জন্য পরীক্ষা পরবর্তী কাউন্সেলিং ফর্ম গোপনীয়

একক আইডি ঃ | | | | | |

১। এইচআইভি পরীক্ষার ফলাফল দেওয়া হয়েছিলঃ দয়া করে কোডে (√) চিহ্ন দিন

☐ এইচআইভি নেগেটিভ অ্যান্টিবডি ☐ এইচআইভি পজেটিভ অ্যান্টিবডি ☐ অনির্ধারিত (অস্পষ্ট ফলাফল)

২। শুধুমাত্র এইচআইভি নেগেটিভ ফলাফলের জন্য ব্যবহার করুন (এই সেশনের জন্য কাউন্সিলরের কাছ থেকে সার্টিফিকেট নিয়ে নিন)

☐ ক্লায়েন্টকে ফলাফল ব্যাখ্যা করা হয়েছে

☐ উইন্ডো পিরিয়ড ও পরবর্তী ঝুঁকিপূর্ণ আচরনের ফলে কি হতে পারে তা বুঝিয়ে বলা হয়েছে

☐ পুনরায় পরীক্ষা করার জন্য উপদেশ দেওয়া হয়েছে

☐ ঝুঁকি হ্রাস করার জন্য আলোচনা করা হয়েছে

৩। শুধুমাত্র অনির্ধারিত (অস্পষ্ট ফলাফল) ফলাফলের জন্য

☐ উইন্ডো পিরিয়ড এর সময় সঞ্চালিত হতে পারে তার সম্ভাবনা ব্যাখ্যা করুন

☐ অরক্ষিত যৌনমিলন অথবা ইনজেক্শান যন্ত্রপাতি শেয়ার করা পরিহার করুন

☐ ১২ সপ্তাহে এই কেন্দ্র থেকে পুনরায় এইচআইভি পরীক্ষা করুন (গর্ভকালীন সময়ের ক্ষেত্রে ৪-৬ সপ্তাহে, যেখানে প্রযোজ্য)

☐ দুঃশ্চিন্তা এবং উদ্বিগ্নতা কমানোর জন্য সহযোগিতামূলক আলোচনা করা

৪। শুধুমাত্র এইচআইভি পজেটিভ ফলাফলের জন্য ব্যবহার করুন

৪.১। পরীক্ষা পরবর্তী কাউন্সিলিং এর সময় কাউন্সিলর এর সার্টিফিকেট

☐ ফলাফল শোনার জন্য কতটুকু প্রস্তুত তা যাচাই করা হয়েছে -- --- --

☐ সেবা গ্রহীতার আত্নহত্যার ঝুঁকি নিরুপন করা হয়েছে -- --- --

☐ পরিবারের সহযোগিতা যাচাই -- --- --

☐ অব্যাহত কাউন্সেলিং সহযোগিতা -- --- --

☐ আর্থিক সহযোগিতা যাচাই -- --- --

☐ চিকিৎসা সহযোগিতা যাচাই -- --- --

☐ মানসিক স্বাস্থ্যসেবার সহযোগিতা -- --- --

☐ ফলোআপ, চিকিৎসা, যত্ন ও সহযোগীতার নেটওয়ার্ক সমন্ধে বিস্তারিত তথ্য আলোচনা -- --- -- -- -- -- -- -- -- -- -- -- -- -- -- --

☐ সঙ্গীর কাছে প্রকাশের কৌশল সম্পর্কে আলোচনা -- --- --

☐ সেবা গ্রহীতার নিরাপদে বাড়ী ফেরা নিশ্চিত করা হয়েছে -- --- --

☐ অন্যান্য (নির্দিষ্ট করুন): -- --- --

৪.২। সেবাগ্রহীতাকে চিকিৎসা, যত্ন ও সহযোগীতার জন্য সেবাদানকারী অংশিদারদের কাছে রেফার করা হয়ে থাকলে:

১। হ্যাঁ ২। না যদি হ্যাঁ, হয় কোথায় (প্রতিষ্ঠানের নাম লিখুন)? -- -- -- -- -- -- -- -- -- -- -- -- -- -- -- -- -- -- -

রেফারের কারন: -- --- --

অন্যন্য মন্তব্য (যদি থাকে): --

--------------------------------- ----------------------------------- --------------------------

কাউন্সিলরের নাম কাউন্সিলরের স্বাক্ষর তারিখ
