# Supplementary material for: Prevalence of HIV, risk behaviours and vulnerabilities of female sex partners of the HIV positive people who inject drugs (PWID) in Dhaka city, Bangladesh
Source: PLoS One. 2023 Jun 5;18(6):e0286673. doi: 10.1371/journal.pone.0286673 (PMC10241362; doi:10.1371/journal.pone.0286673)
Supplement: Attachment — Submitted filename: Response to Reviewers.docx [file pone.0286673.s003.docx]

**Reviewer #1:**

• Suggestion to revise the title: Prevalence of HIV, risk behaviours and vulnerabilities of female sexual partners of HIV positive people who inject drugs in Dhaka, Bangladesh

RESPONSE: The title has been revised.

• From the outset, please be clear that this study was done on spouses of male injection drug users. Your paper is still valuable even if most (82.4%) of the study participants were spouses.

RESPONSE: In our study, 227 female sex partners of PWID were included. Of 227, 136 (59.9%) were wives, 66 (29.1%) were female sex workers and 25 were other female sex partners (11.0%). Therefore, this study was done not only on spouses but also among other female sex partners of HIV positive PWID.

**Abstract**
• People who inject drugs (PWID) is more renown and globally used term.

RESPONSE: MWID has been replaced with PWID in the whole manuscript.

• Please write IBBS for the first time in full.

RESPONSE: Full abbreviation of IBBS has been provided when it appears for the first time.

• A more focused last sentence of the background section could be: This paper describes the HIV prevalence, risk behaviours, and vulnerabilities of PWIDs and their sexual partners.

RESPONSE: Out study was conducted among female sex partners of HIV positive PWIDs. Female sex partners were tested for HIV and face to face interview was conducted with them. HIV positive male PWIDs were not the target population in this study and hence no information was taken from them. They were just the entry points to the participants of our study.

• Please revise methods: This was a cross-sectional study conducted among 227 female sexual partners of HIV-positive MWID or PWID in Dhaka, Bangladesh, in 2019, adopting a take-all sampling technique.

RESPONSE: The method section has been revised as suggested.

• Please write STI in full for the first time.

RESPONSE: Full abbreviation of STI has been provided when it appears for the first time.

• What does “receive” in this sentence mean: Forty three percent (95% CI: 36.4-49.3) received HIV testing services and knew the result during last year. Please rephrase if this was part of outreach through the DICs.

RESPONSE: This was a routine HIV testing services by the PWID DICs/CDICs and this has been rephrased in the manuscript.

• One-third never received any services. Are these HIV prevention services?

RESPONSE: Yes, this has been rephrased in the manuscript.

• Please stick to the same pattern as you have done in previous sentences, which is to write in words instead of numbers after a full stop. 46% (95% CI: 39.3-52.6) reported been beaten and 20.2% (95% CI: 15.3-26.1) been raped during last year.

RESPONSE: This line has been rephrased in the abstract.

• Husbands? Most of the episodes of beating and rape were perpetuated by HIV-positive husbands (61-67%). If this is about spouses, then please revise the title.

RESPONSE: This issue has been explained with data in the results section of Physical and sexual violence, and other vulnerabilities.

• The conclusion does not match the findings. Please revise.

RESPONSE: The conclusion has been revised as suggested.

Introduction
• As mentioned earlier, PWID is a more common term used globally.

RESPONSE: All MWIDs have been replaced with PWIDs in the manuscript.

• Line 56: please revise to has risen from was risen.

RESPONSE: ‘was risen’ has been changed to ‘has risen’.

• Lines 70-74 need to be revised. Please only mention the rationale and objective here.
RESPONSE: This has been revised as suggested.

Methods
• Please revise the methods in the following pattern: study type, population, sample size, sampling, and timeline of data collection. This is how the methods section should ideally start. The rest of the sections can follow.

RESPONSE: The method section has been revised as suggested.

• Is there a difference between a drop in centre and a comprehensive drop in centre?
RESPONSE: Drop in centres (DICs) and Comprehensive DICs both provide HIV prevention services to PWIDs. In addition, CDICs provide ART services to the HIV positive PWIDs.

Results
• The narration in the results section needs to be trimmed. It is far too long, and the reader gets lost. Mention key findings and refer to the tables.

RESPONSE: Results sections have been shortened as suggested.

• Please give serious thought to merging variables of tables 3-6 into one table.

RESPONSE: Tables 2-3 refers to risk behaviours and tables 4-6 refers to vulnerability. If we merge tables 4-6, the table will become too long. Therefore, we kept tables 4-6 separated.

• The mean age of 15.6 years; is the time of being married or the mean age of the respondent at the time of marriage?

RESPONSE: It is the mean age of the respondent at the time of marriage, the line has been revised in the manuscript.

• The median number can be deleted from the table.

RESPONSE: The second reviewer suggested to delete mean values and keep the median values. In that case, I am confused what to do?

• Line 187: this is the spouse/sexual partner of the drug user? Please clarify.

RESPONSE: This is the combined of wives/FSWs/other female sexual partners.

• It is a suggestion to highlight condom use in the last sexual act (22.7%) prominently, as this is far more important than consistent condom use in the last year, which has a high probability of recall bias.
RESPONSE: We have revised the discussion section on condom use as suggested.

Conclusion
• Please reduce the conclusion to two to three sentences.

RESPONSE: The conclusion has been shortened as suggested.

**Reviewer #2:**

Thanks for the review. The article describes prevalence and risk behaviours of partners of HIV positive men who inject drugs.

General comment: The language requires editing. e.g Professional sex workers instead of " selling sex" among others.

RESPONSE: We have added “Professional sex workers” with selling sex within brackets.

Comment 1:
"Four new cases are on ART and 3 are still no trace". Does it mean that they have been lost to follow up?
RESPONSE: Yes, mentioned in the manuscript.

Comment 2: Please share response rate to questionnaires in first paragraph of results. Numbers keep changing in all tables and there is significant attrition of numbers while in text only percentages have been reported.
RESPONSE: Response rate is provided before the result section started.

Comment 3: Would be best to report medians and IQRs throughout rather than reporting means and medians both.

RESPONSE: The first reviewer suggested to delete medians and keep the mean values. In that case, I am confused what to do?

Comment 4: How were 95% CI computed and what was the point estimate. There is no mention in methods regarding what statistical tests have been used.
RESPONSE: This has been explained in the Data management and analysis section.

Comment 5: Subheadings in discussion are not required

RESPONSE: All sub headings are removed in the discussion section.

Comment 6: The discussion lacks appropriate comparisons with local and regional settings. Should elaborate why the uptake was not sufficient and how that was comparable to other countries. In some parts discussion seems like an elaborate write up of results and needs to be re-written as discussion especially the paragraphs on uptake and vulnerabilities.

RESPONSE: We have tried our best to provide information from regional and other countries to compare the findings with our study. We have also shortened the discussion and re-written where needed. In the discussion, we have also mentioned that the uptake of HIV testing and prevention services were low due to lack of designated field staff.
